# Two-Stage Probabilistic Classification of Sperm Identity and Morphology

**DOI:** 10.3390/diagnostics16203390

**Published:** 2026-10-18

**Authors:** Emrah Zayman, Feyza İnceoğlu

**Affiliations:** 1 Department of Histology and Embryology, Faculty of Medicine, Malatya Turgut Özal University, Malatya 44210, Türkiye; emrah.zayman@ozal.edu.tr; 2 Department of Biostatistics and Medical Informatics, Faculty of Medicine, Malatya Turgut Özal University, Malatya 44210, Türkiye

**Keywords:** sperm morphology, andrology, artificial intelligence, clinical decision support, conformal prediction

## Abstract

**Background/Objectives:** From a histological and embryological perspective, sperm identification and morphological classification are distinct assessment tasks. This study compares image-level error distributions and review-referral outcomes between separately trained conditional components and direct three-class learning. **Methods:** Following duplicate-image and label audits, 2947 images from the Sperm Morphology Image Dataset (SMIDS) were analyzed without changing the source labels. Logistic regression models using frozen EfficientNet-B0 features were evaluated using five outer and three inner cross-validation folds that preserved 2942 similarity groups. Final class probabilities, component probabilities, and outcomes at matched review volumes were compared. **Results:** Overall accuracy was 83.98% for the two-stage model and 84.80% for the direct model. Misclassification of non-sperm images as sperm decreased from 94 (direct) to 77 (two stage), whereas misclassification of true sperm images as non-sperm increased from 82 to 141. When each model referred 973 images for review, accuracy among the remaining 1974 images was 93.67% and 94.48%, respectively. **Conclusions:** The two models differed in the trade-off between erroneously including non-sperm material and excluding assessable sperm images. Overall and selective accuracy were higher for the direct model. The study provides a framework for jointly evaluating these two error directions on the same test images and at matched review volumes in preliminary histomorphological assessment.

## 1. Introduction

**Figure 1:**
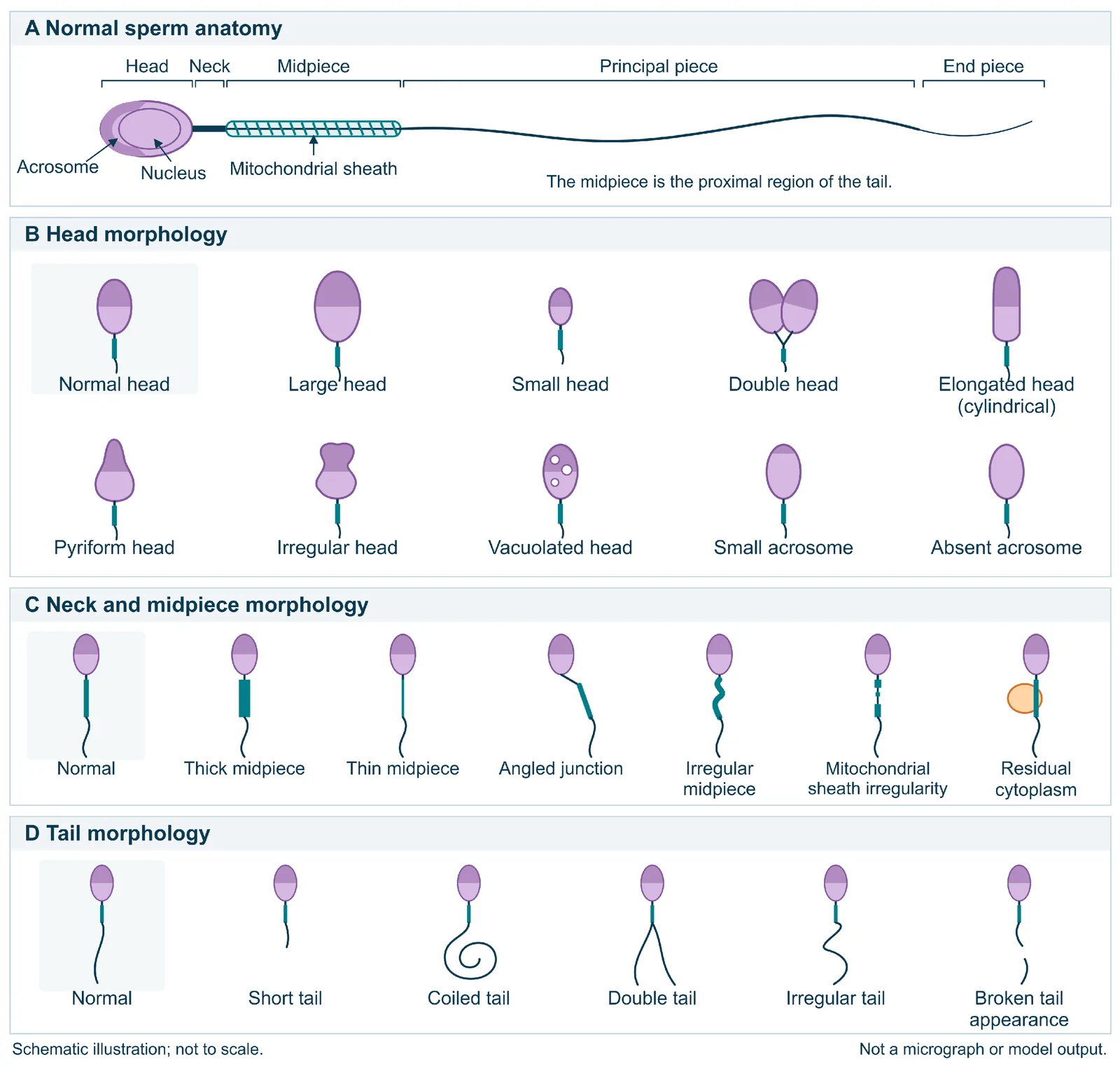
Normal sperm anatomy and examples of regional morphological appearances. (**A**) Schematic arrangement of the head, neck, midpiece, principal piece, and end piece, together with the acrosome, nucleus, and mitochondrial sheath. (**B**) Normal reference head and large, small, double, elongated, pyriform, irregular, and vacuolated heads, together with small or absent acrosomal appearances. (**C**) Normal reference and thick or thin midpieces, an angulated head–tail junction, an irregular midpiece, an irregular mitochondrial sheath, and residual cytoplasm. (**D**) Normal reference and short, bent, double, irregular, and broken tails. The anatomical framework follows WHO criteria [5], Auger et al. [3], and Teves and Roldan [1]. Examples with light backgrounds indicate normal references. Drawings are not to scale and are neither Sperm Morphology Image Dataset (SMIDS) micrographs nor model outputs.

A spermatozoon is a haploid reproductive cell that acquires, through spermatogenesis and spermiogenesis, the structural characteristics required to deliver paternal genetic material to the oocyte. The head of the mature cell contains a condensed nucleus and an acrosome covering the anterior nuclear region. The neck connects the head to the flagellum; the midpiece of the flagellum contains the mitochondrial sheath and continues into the principal piece and end piece [1]. Morphological assessment considers the head contour, head–tail junction, midpiece, tail, and excess residual cytoplasm together; a normally shaped head alone does not establish normal morphology of the entire cell [2,3,4]. According to World Health Organization (WHO) criteria, the head contour should be smooth, the acrosome should cover approximately 40–70% of the head area, and large vacuoles should be absent. There should be no more than two small vacuoles, together occupying no more than one-fifth of the head area, and no vacuoles should be present in the postacrosomal region [5]. The midpiece should be regular and aligned with the long axis of the head [2,3]. Light and dark regions within the head and proximal thickening should be interpreted within the limits of image resolution; specific defects or chromatin damage require additional structural verification [6]. Morphology describes visible cellular characteristics; it does not directly measure fertilizing capacity and should be interpreted alongside other semen findings and clinical information [7,8] (Figure 1).

In andrology, the field concerned with male reproductive health, semen examination links clinical assessment to reproductive laboratory findings [9]. The European Society of Human Reproduction and Embryology (ESHRE) recommendations for in vitro fertilization (IVF) laboratories emphasize the need for trained personnel and quality assurance throughout sample handling, assessment, and record keeping [10]. Semen analysis evaluates macroscopic ejaculate characteristics, sperm count, motility, and morphology under standardized conditions [11]. The sixth edition of the *WHO Laboratory Manual for the Examination and Processing of Human Semen* provides the fundamental framework for standardized procedures and clinical interpretation of reference distributions [5,12]. Macroscopic assessment includes liquefaction, appearance, volume, viscosity, and acidity or alkalinity (pH). Microscopic assessment includes concentration, total sperm count, motility, vitality, and morphology; agglutination and non-sperm cellular elements are also recorded [5]. Because staining can affect measured sperm head dimensions, morphological appearances should be interpreted in the context of preparation conditions [13].

Campbell et al. derived reference distributions from men whose partners conceived within 12 months; the fifth percentile for morphologically normal sperm was 4% [14]. This reference value is not a standalone cutoff distinguishing fertile from infertile men [14]. Morphology should be interpreted alongside other semen findings and the clinical context [7,11]. Classification of a single image, the proportion of normal forms in an ejaculate, and a clinical diagnosis of infertility therefore represent different levels of assessment [8,11].

From a histological and embryological perspective, automated classification should preserve the visible anatomical basis of both cell identification and morphological interpretation [3]. Expert-defined structural labels are important for linking image analysis to these assessment tasks [4,15].

In sperm image analysis, shape and texture features, convolutional neural networks (CNNs), and transfer learning have been used for various classification tasks [16]. Chang et al. presented a reference dataset for sperm head morphology [17], while Javadi and Mirroshandel investigated deep learning for different sperm assessment tasks [18]. Other approaches include the genetic neural architecture search investigated by Miahi et al. [19], integrated sperm detection and classification [20], comparison of convolutional networks with vision transformers [21], attention-based deep feature processing [22], and the comparison by AlSaad et al. of vision transformers and visual foundation models on SMIDS, HuSHeM, and Hi-LabSpermMorpho [23]. However, sperm head subtypes, whole-cell normal–abnormal morphology, and sperm–non-sperm discrimination are distinct labeling targets [4,17,20]. Each model’s contribution should therefore be interpreted within its anatomical scope and decision task.

Previous studies have combined sperm detection and classification, developed deep feature representations, or used two-stage structures based on anatomical defect groups [20,22,24]. The question addressed here is which decisions change when sperm identity and conditional morphology are learned separately using shared image features and the same test partitions. In this context, inclusion of non-sperm material in morphological classes and exclusion of true sperm images are distinct errors in preliminary histomorphological assessment. Examining both reveals the relationship between correct cell identification and preservation of assessable sperm cells.

The original contribution of this study is to examine the effects of separate learning on cell identity and morphology decisions within the same experimental design. These effects were evaluated through bidirectional identity errors, component probabilities compared on the same tasks, and outcomes at matched review volumes. A frozen image representation, shared test partitions preserving similarity groups, and record-level pairing allow comparison of two specific learning implementations. Source expert labels were retained, while cellular examples helped relate numerical decisions to visible anatomical characteristics. From a histological and embryological perspective, this design links model evaluation to the question of which images enter morphological assessment and which are excluded.

## 2. Materials and Methods

### 2.1. Study Design and Data Source

This image-level secondary analysis used the publicly available Sperm Morphology Image Dataset (SMIDS) archive. A two-stage probabilistic model was developed to learn sperm identity and morphological class in separate components. The first component used all training classes to learn the probability of sperm identity, whereas the second used only training images labeled as normal or abnormal sperm to learn morphological class probabilities conditional on sperm identity. At test time, both components received the same image representation. Their calibrated probabilities were combined without a hard gate at the first stage to calculate final probabilities for the normal, abnormal, and non-sperm classes. The final label was the class with the highest final probability. Using the same image representation and the same outer-test partitions, a direct three-class model that predicted the classes served as the comparator.

SMIDS version 1, published on Mendeley Data, was used [25]. The archive contains 3000 images: 1021 normal, 1005 abnormal, and 974 non-sperm images. Files are stored in bitmap (BMP) format with red–green–blue (RGB) color channels. The source study describes smartphone-based microscopic image acquisition, segmentation of candidate sperm regions, and expert classification of images into normal, abnormal, or non-sperm reference classes [20]. No new samples were collected, and no additional segmentation was performed in this study.

Because an image may contain multiple heads or intersecting tails, sample size was defined as the number of images rather than the number of independent spermatozoa or patients. The primary endpoint was classification of normal, abnormal, and non-sperm images according to the source labels. Sperm identity and normal–abnormal morphology within reference sperm images were also evaluated at the component level.

### 2.2. Ethics and Use of Reference Labels

The SMIDS source study reported ethical approval from the ethics committee of Istanbul University Faculty of Medicine, compliance with the 1964 Declaration of Helsinki and its subsequent amendments or equivalent standards, and informed consent from all participants [20]. No new semen samples were collected in this secondary analysis; images from the published archive were used.

The source normal, abnormal, and non-sperm labels were retained as the reference standard. This secondary analysis involved no new expert annotations, consensus sessions, or recorded clinical assessments. The abnormal class was retained as a general morphological category, distinct from labels for regional head, neck, midpiece, or tail defects. Histomorphological descriptions and anatomical arrows were provided as explanatory illustrations rather than as a reference standard or independent expert annotations.

### 2.3. Data Consistency and Similar-Image Audit

Files with identical pixel content were identified using a SHA-256 hash that included both image dimensions and the pixel array after RGB conversion. One copy of each same-label duplicate was retained; all copies with conflicting labels were excluded. Near-duplicate candidates were screened using a maximum Hamming distance of 2 between 64-bit perceptual hashes. Pairs with a structural similarity index of at least 0.995 were assigned to the same group. Grouping used computational thresholds without new expert labeling. Procedural details are provided in Supplementary Section S1.7; image identifiers and fold assignments are included in the reproducibility package.

The audit removed 45 redundant same-label copies and eight files belonging to four conflicting-label pairs, leaving 2947 images. Five near-duplicate pairs were grouped, yielding 2942 image-similarity groups (Table 1).

### 2.4. Image Preprocessing and Models

Images were read as RGB and fitted to a 224 × 224 canvas while preserving their aspect ratios. Empty areas were filled with the median image color, and ImageNet color normalization was applied. Means and standard deviations for feature standardization were calculated within each training partition.

In the main analysis, EfficientNet-B0 pretrained on ImageNet provided the shared image representation for both learning structures; its final classification layer was removed, and it was used as a frozen feature extractor to obtain 1280 features per image [26]. Features were standardized using StandardScaler and entered into logistic regression with balanced class weights. Weighting was important for the first component because 1974 sperm and 973 non-sperm images formed an approximately 2:1 distribution, whereas class sizes were similar in the three-class and morphology tasks. The same weighting rule was used for all components; its effect was examined separately by retraining without weights on the same folds (Table 2; Supplementary Section S6.3). Network weights remained frozen throughout all comparisons, and training was restricted to regularized classifiers fitted to the extracted features. This allowed the effect of separating learning targets to be examined under a shared image representation.

Logistic regression models were implemented using scikit-learn version 1.9.0, with the limited-memory Broyden–Fletcher–Goldfarb–Shanno (L-BFGS; lbfgs in the software) solver for the direct three-class model and the liblinear solver for the binary components. Analyses were conducted in Python 3.12. Additional software versions are provided in Supplementary Section S1.6.

Generative artificial intelligence (AI) use. OpenAI ChatGPT and Codex were used to support language editing, Turkish-to-English translation, refinement of analysis codes, and the preparation of illustrative schemas and figure captions. In Codex, the GPT-5 Sol model was utilized with the reasoning level set to “High”. The schemas are illustrative in nature and were not used to replace source micrographs, source class labels, or training and test data. The authors have reviewed and edited the generated content and take full responsibility for the scientific accuracy of the final text, codes, results, and interpretations.

Deep features were used to predict the source normal, abnormal, and non-sperm classes. Histomorphological assessment described cellular structures discernible in the images; organelle-level quantitative measurements were treated as a separate assessment target.

### 2.5. Calculation of the Two-Stage Decision

Stage 1 estimated the probability of sperm identity, p(S), for each image. Stage 2 was trained only on images labeled as normal or abnormal and estimated the probability of abnormality conditional on sperm identity, p(A|S). The Stage 2 output represents morphology conditional on sperm identity and contributes to the final abnormal class probability through multiplication by the sperm-identity probability.

**Figure 2:**
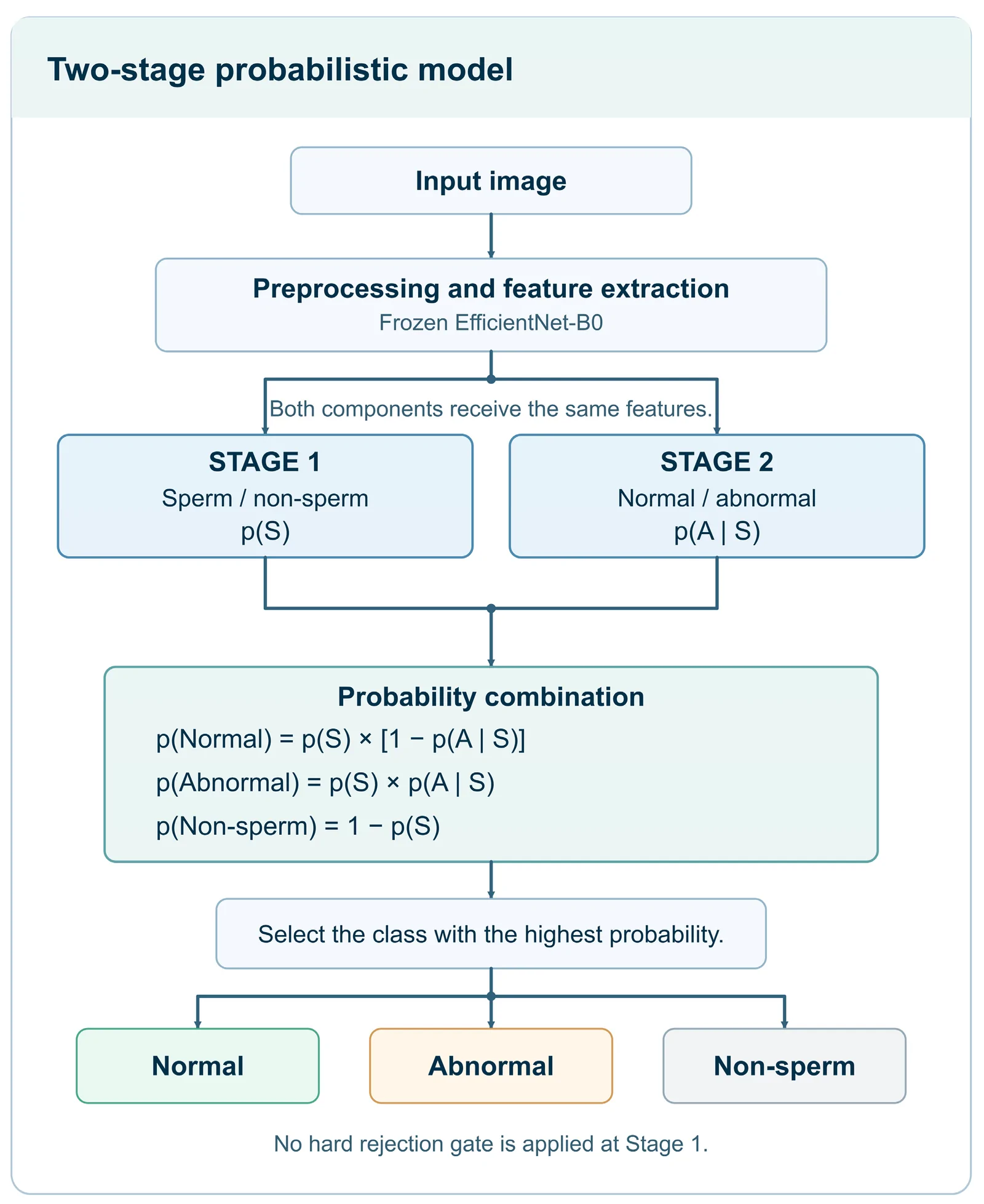
Decision flow of the two-stage probabilistic model. Features extracted from the same image are supplied to the sperm-identity and conditional-morphology components; calibrated probabilities are combined to determine the final class. The second component is trained only on images labeled as normal or abnormal sperm. No hard gate is applied at the first stage. p(S) denotes the probability of sperm identity, and p(A|S) denotes the abnormal class probability conditional on sperm identity.

Final class probabilities were calculated as p(N) = p(S) × [1 − p(A|S)], p(A) = p(S) × p(A|S), and p(D) = 1 − p(S), where N, A, and D denote the normal, abnormal, and non-sperm classes, respectively. The class with the highest final probability was selected. No hard gate was applied before Stage 2. The 0.50 threshold was used only to evaluate each binary component separately; Stage 2 was evaluated on all 1974 reference sperm images (Figure 2). Both final sperm-class probabilities are bounded above by p(S). When p(S) < 0.50, the non-sperm probability exceeds both sperm-class probabilities regardless of the conditional-morphology output.

For the direct model, p_d(S) = p_d(N) + p_d(A) and p_d(A|S) = p_d(A)/[p_d(N) + p_d(A)] can also be calculated. This shared probability representation allows direct comparison of the two learning structures on the sperm-identity and conditional-morphology tasks. These derived probabilities were compared with the separately learned components on the same outer-test images and with identical task denominators (Supplementary Section S6.1).

### 2.6. Model Validation and Evaluation

Five outer and three inner cross-validation folds were used, preserving similarity groups and accounting for class distribution. Standardization and model fitting were performed separately within each inner-training partition. The regularization parameter C was selected from 0.01, 0.1, and 1 according to inner-validation balanced accuracy; when two values yielded equal balanced accuracy, the smaller C (stronger regularization) was preferred. Temperature calibration used out-of-fold predictions for the selected C; temperature was obtained by bounded one-dimensional minimization of log loss over the interval from 0.25 to 4.0 [27]. Outer-test data were excluded from these procedures. Each image was evaluated once in an outer-test fold; the results represent image group-based internal validation within the same archive. Details are provided in Supplementary Sections S1.5 and S2.1.

Performance was evaluated using accuracy, balanced accuracy, precision, sensitivity, F1 score, macro-F1, and the Matthews correlation coefficient (MCC). Curve and calibration analyses assessed discrimination and probability agreement. Metric definitions and detailed procedures are provided in Supplementary Sections S1.3 and S1.5, and curve and calibration results are provided in Supplementary Sections S3 and S4.

Predictions were paired on the same outer-test images. The 95% confidence intervals were calculated by bootstrap resampling of similarity groups, and differences in correct and incorrect decisions were evaluated using the two-sided exact McNemar test. Statistical evaluation was based on the available image-similarity groups. Performance measures were interpreted alongside error counts and directions in the paired records (Supplementary Sections S1.5 and S2).

In a supplementary threshold analysis, the sperm-identity threshold was varied from 0.00 to 1.00 using stored outer-test probabilities. Erroneous acceptance of non-sperm images and exclusion of reference sperm images were calculated for both models. This binary decision rule was evaluated separately from the three-class rule of the main experiment, which selects the class with the highest final probability. Methodological details are provided in Supplementary Section S2.4.

Visible head, junction, and tail characteristics were described in three purposively selected test images whose source labels remained unchanged. Stage-specific gradient-weighted class activation maps (Grad-CAM) visualized contributions to the decision score [28]. Maps were presented separately from the original micrograph within the same spatial frame. These panels were used to examine visible cell characteristics together with stage-specific classification responses (Section 3.6; Supplementary Sections S1.1 and S1.2). To determine how common the frame-edge pattern was, the sperm-identity map was calculated for every outer-test image and the conditional-normal map for every reference sperm image (n = 1974), and whether the highest-contributing cell lay in the outer ring of the 7 × 7 map (24 of 49 cells) was recorded. Rates are reported with 95% Wilson intervals, excluding empty maps from the denominator (Table S1).

Confidence threshold-based selective evaluation and a supplementary split-conformal experiment with a separate calibration partition were conducted [29]. Selective evaluation examined accuracy of retained decisions alongside coverage [30]. Images below the confidence threshold were designated as review candidates. Supplementary Materials analyses used outer-test probabilities retained from the main experiment. The direct and two-stage models were compared by ranking confidence based on final class probabilities and retaining decisions for the same number of images. Component probabilities derived from the direct model were also compared with separately learned components on the same tasks. Review volume and errors remaining outside review were reported (Supplementary Sections S1.8 and S6.1–S6.3). In the conformal experiment, retrained with a separate calibration partition, multi-label sets were designated as review candidates; these results were reported separately from the main experiment.

With the B0-based main analysis retained, sensitivity to feature-extractor choice was examined in supplementary experiments using B1 and B2. The same images, 224 × 224 preprocessing, and validation partitions were used. Methods and paired statistical comparisons are provided in Supplementary Section S6.6.

## 3. Results

### 3.1. Images Included in the Analysis

The analysis included 2947 images: 1002 normal, 972 abnormal, and 973 non-sperm images. Each image was evaluated once in an outer-test fold. Fold-level details are provided in Table S2.

### 3.2. Overall Performance of the Two-Stage Model

The two-stage model correctly classified 2475 of 2947 images (83.98%), whereas the direct three-class model correctly classified 2499 (84.80%). Balanced accuracy was 84.00% and 84.81%, and macro-F1 was 0.839 and 0.848, respectively (Table 3). These values describe the combined final decision rather than the separate results of the sperm-identity and morphology components.

The 95% confidence interval for balanced accuracy was 82.74–85.25% for the two-stage model and 83.54–86.08% for the direct three-class model. The paired difference was −0.81 percentage points (95% confidence interval, −1.55 to −0.08; Table S3). The small difference favoring the direct three-class model (0.8 percentage points) was supported by the exact McNemar test (*p* = 0.0369); 73 images were classified correctly only by the direct three-class model and 49 only by the two-stage model.

### 3.3. Separate Results of the Sperm-Identity and Morphology Components

Stage 1 correctly identified 1851 of 1974 sperm images and 890 of 973 non-sperm images. Sensitivity was 93.77%, specificity was 91.47%, and accuracy was 93.01% (Table 4). These results describe recognition of a structure as sperm independently of normal–abnormal morphological classification.

The two components were evaluated separately at a probability threshold of 0.50. For Stage 2, the denominator comprised all 1974 images with source sperm labels regardless of the Stage 1 acceptance decision.

The second component correctly classified 821 of 972 abnormal images and 866 of 1002 normal images; accuracy was 85.46%, sensitivity was 84.47%, and specificity was 86.43%. Component results were reported for their respective tasks and reference sets. Abnormal sensitivity was 84.47% in reference sperm images and 77.57% in the final three-class decision after combination with the sperm-identity probability.

Accuracy for the sperm-identity probability derived from the direct model was 94.06%, compared with 93.01% for the separately learned sperm component (n = 2947). In the reference sperm subset, derived conditional-morphology accuracy was 85.21%, compared with 85.46% for the separately learned component (n = 1974). The comparison on identical tasks and denominators enabled comparison of sperm-identity and conditional-morphology decisions between the two learning structures (Supplementary Section S6.1; Table S7).

### 3.4. Class-Specific Results and Error Directions

In final class decisions, sensitivity of the two-stage model for normal, abnormal, and non-sperm images was 82.34%, 77.57%, and 92.09%, respectively. Precision for normal and abnormal decisions increased relative to the direct model, whereas sensitivity for these classes decreased. In the non-sperm class, higher sensitivity was accompanied by lower precision (Table 5).

**Figure 3:**
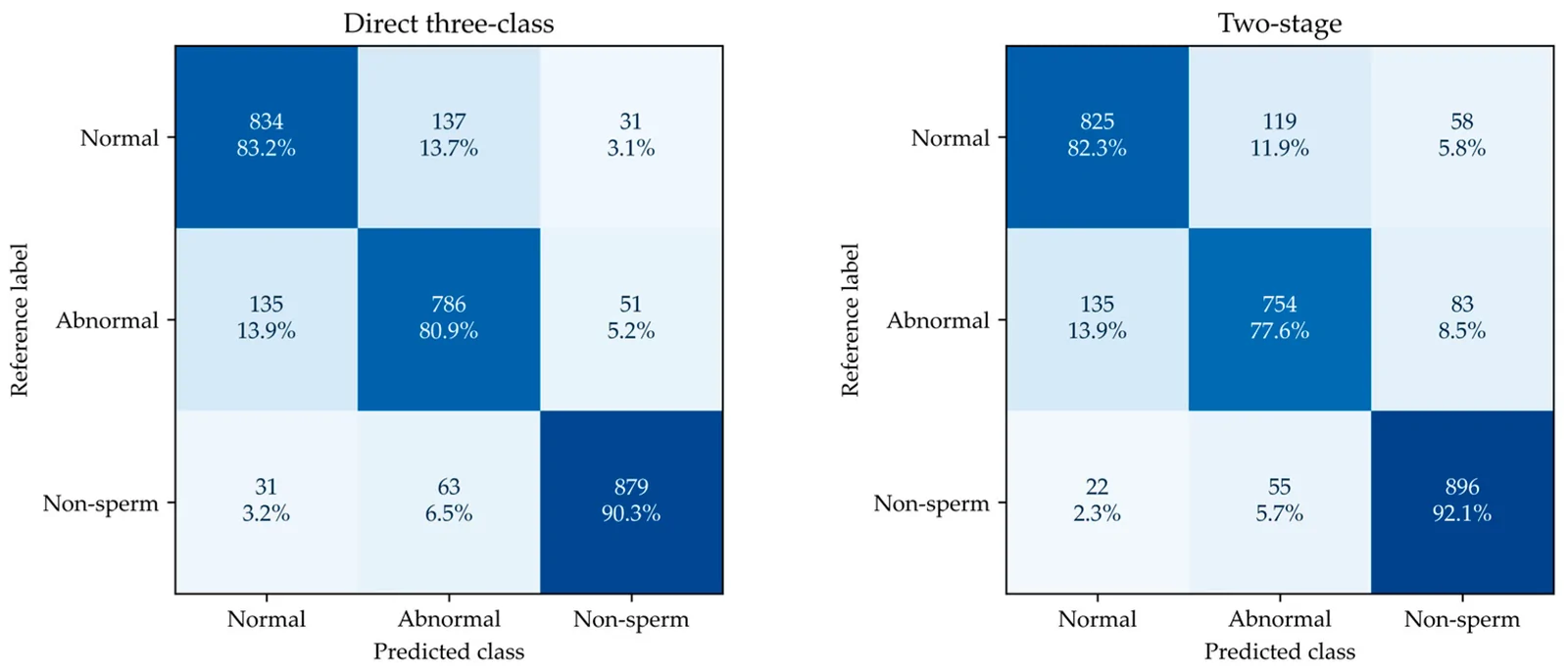
Confusion matrices for the direct three-class and two-stage models. Rows indicate reference classes, and columns indicate predicted classes. Cells show image counts and row percentages.

In three-class decisions based on the highest final class probability, moving from the direct model to the two-stage model reduced non-sperm-to-sperm errors from 94 to 77 while increasing reference sperm images assigned to the non-sperm class from 82 to 141 (Figure 3). The net change was a reduction of 17 errors in the first direction and an increase of 59 errors in the reverse direction. In preliminary histomorphological assessment, this finding describes the trade-off between excluding non-sperm material and preserving assessable sperm cells. Supplementary results obtained by directly thresholding sperm-identity probability are reported separately in Section 3.8.

Among normal images, erroneous abnormal decisions decreased from 137 to 119, whereas normal-to-non-sperm decisions increased from 31 to 58. Abnormal-to-normal errors remained at 135 for both models; abnormal-to-non-sperm errors increased from 51 to 83. The models disagreed on 136 images (4.61%; Supplementary Section S2.2).

### 3.5. Confidence Threshold and Images Referred for Review

**Figure 4:**
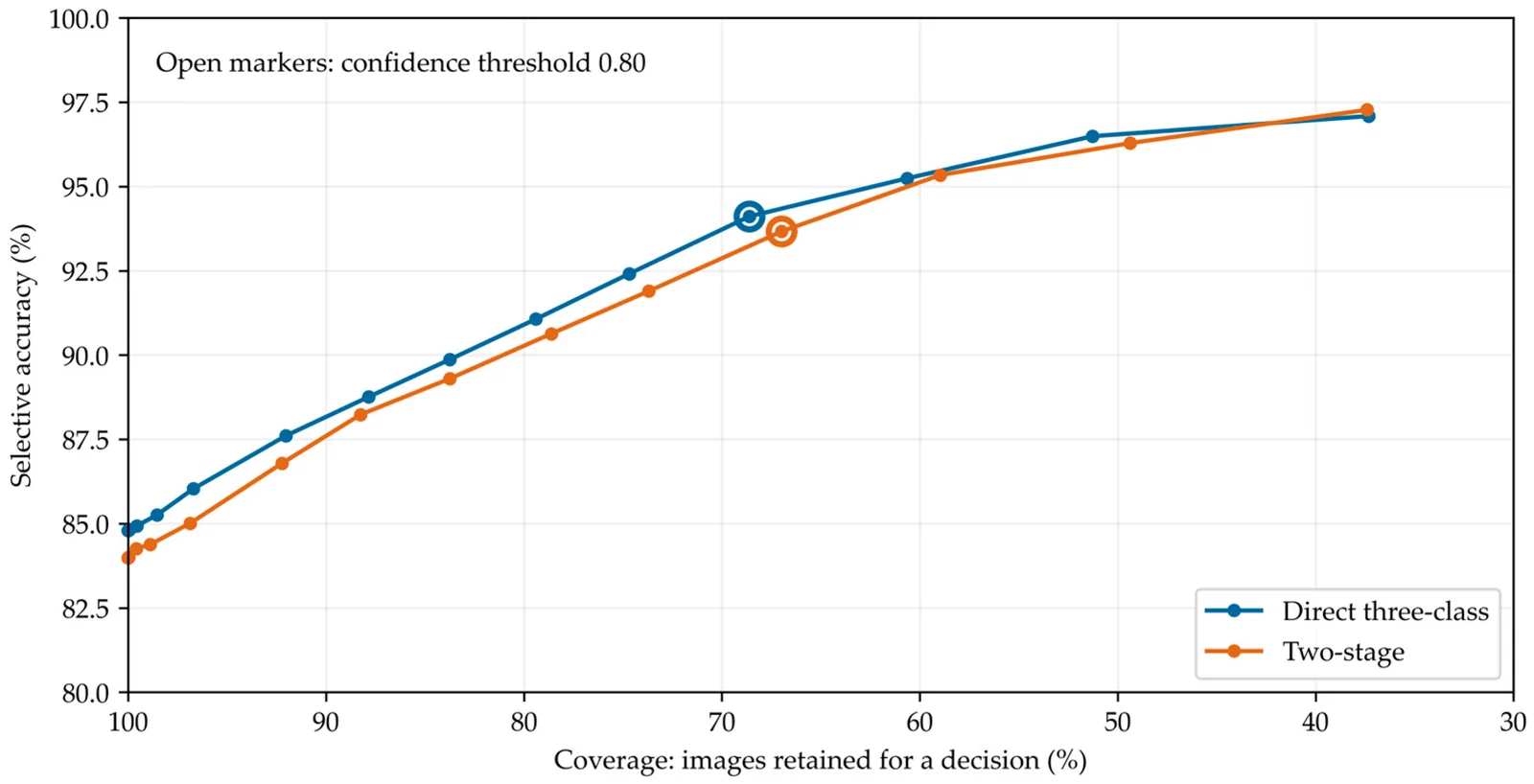
Coverage and selective accuracy across confidence thresholds. The horizontal axis indicates decreasing coverage; open markers denote the 0.80 threshold. Curves for both models were calculated from pooled outer-test predictions of the main experiment.

At a confidence threshold of 0.80, the two-stage model retained 1974 images (66.98% coverage); with 1849 correct and 125 incorrect decisions, selective accuracy was 93.67%, and 973 images remained for review. The direct three-class model retained 2022 images (68.61% coverage); selective accuracy was 94.11%, and 925 images remained for review (Figure 4). This threshold is a research operating point for the confidence–coverage relationship; selecting a clinical threshold requires prospective evaluation under the target laboratory’s conditions.

Because the same threshold retained decisions for different numbers of images, a matched review volume comparison was also performed. When both models referred 973 images for review, accuracy among the remaining 1974 images was 94.48% for the direct model (109 errors) and 93.67% for the two-stage model (125 errors). In these subsets, non-sperm-to-sperm error counts were 25 and 19, and sperm-to-non-sperm error counts were 20 and 50, respectively. At matched review volume, the direct model produced fewer total errors, whereas the two-stage model produced fewer erroneous assignments in the non-sperm-to-sperm direction. Other coverage levels are reported in Supplementary Section S6.2, Table S8, and Figure S5.

### 3.6. Cell-Level Morphological Assessment

**Figure 5:**
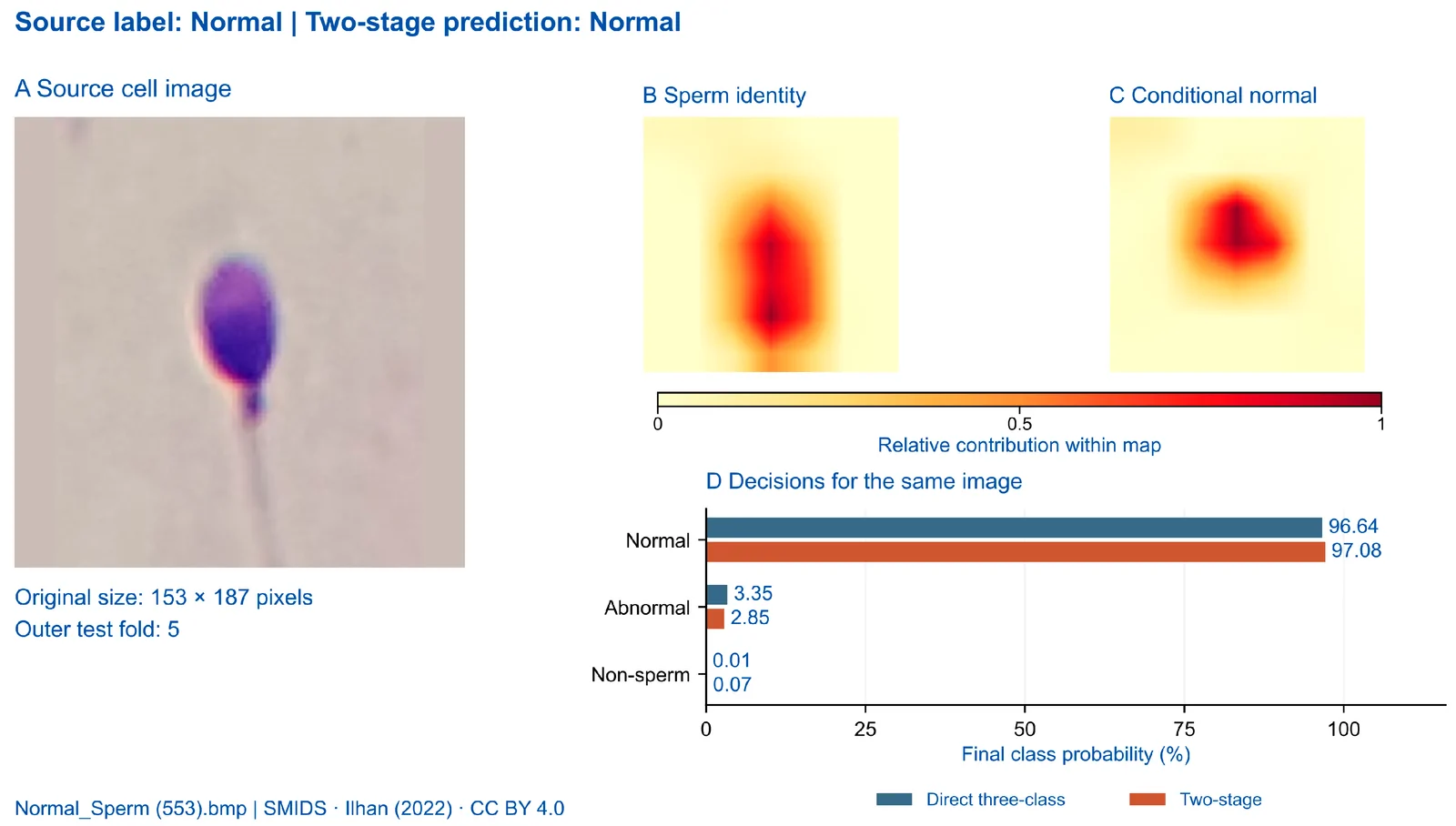
Normal_Sperm (553).bmp. (**A**) Source cell image padded to a square frame. (**B**) Sperm-identity and (**C**) conditional-normal Grad-CAM maps. (**D**) Final class probabilities from both models for the same image. Maps are presented in the same spatial frame as the micrograph. The color scale indicates relative contribution to the target class score and is normalized to each map’s own positive maximum. Source: SMIDS, CC BY 4.0 [25].

**Figure 6:**
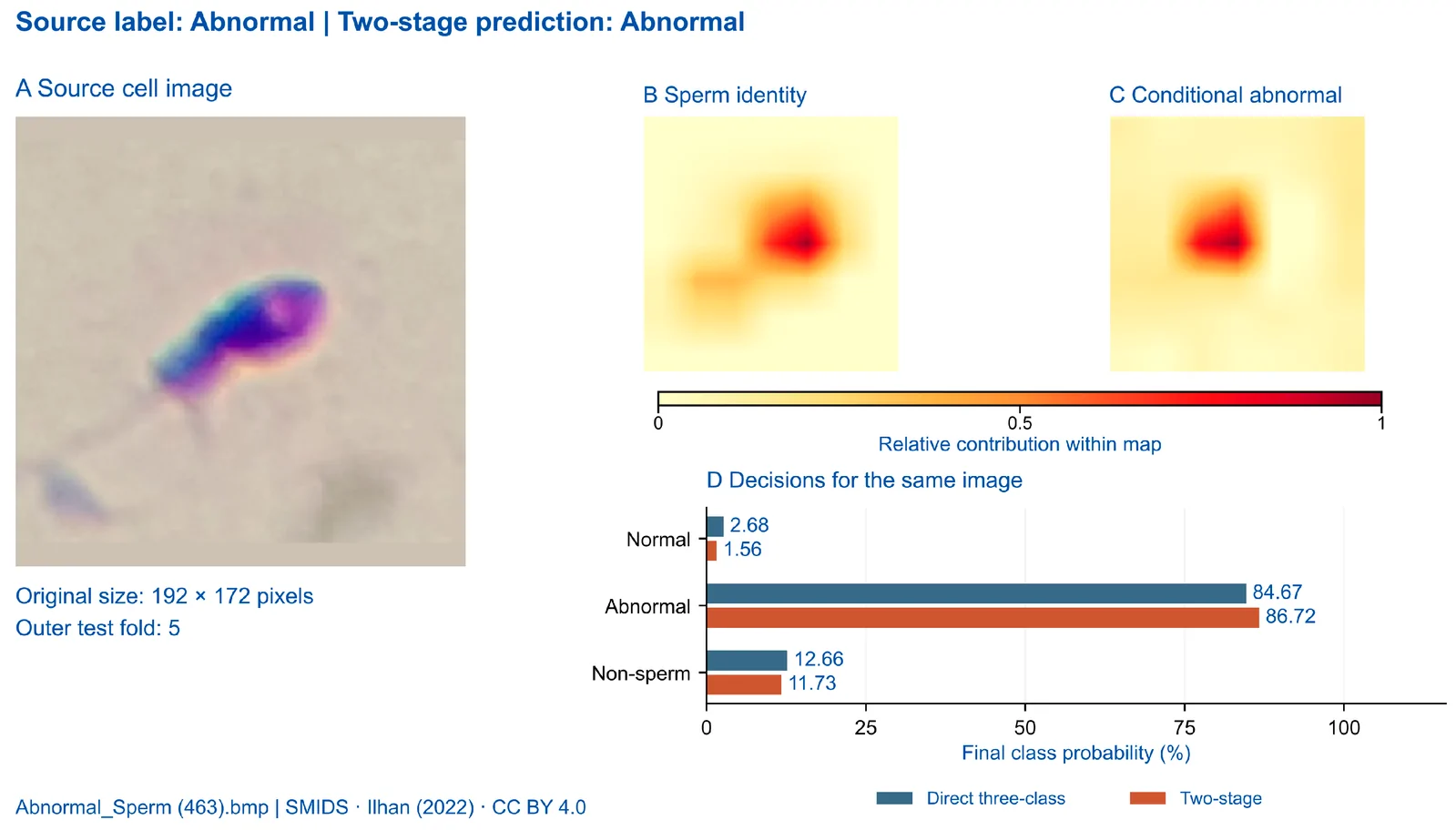
Abnormal_Sperm (463).bmp. (**A**) Source cell image padded to a square frame. (**B**) Sperm-identity and (**C**) conditional-abnormal Grad-CAM maps. (**D**) Final class probabilities from both models for the same image. Maps are presented in the same spatial frame as the micrograph. The color scale indicates relative contribution to the target class score and is normalized to each map’s own positive maximum. Source: SMIDS, CC BY 4.0 [25].

The normal-labeled example showed an oval head profile and a visible head–tail junction. The sperm-identity probability was 99.93%, the conditional-normal probability was 97.15%, and the final normal probability was 97.08% (Figure 5). An abnormal reference image showed a densely stained head, proximal thickening, and a visible low-contrast tail segment; the sperm-identity probability was 88.27%, the conditional-abnormal probability was 98.23%, and the final abnormal probability was 86.72% (Figure 6). Both models agreed with the source labels for these two images.

**Figure 7:**
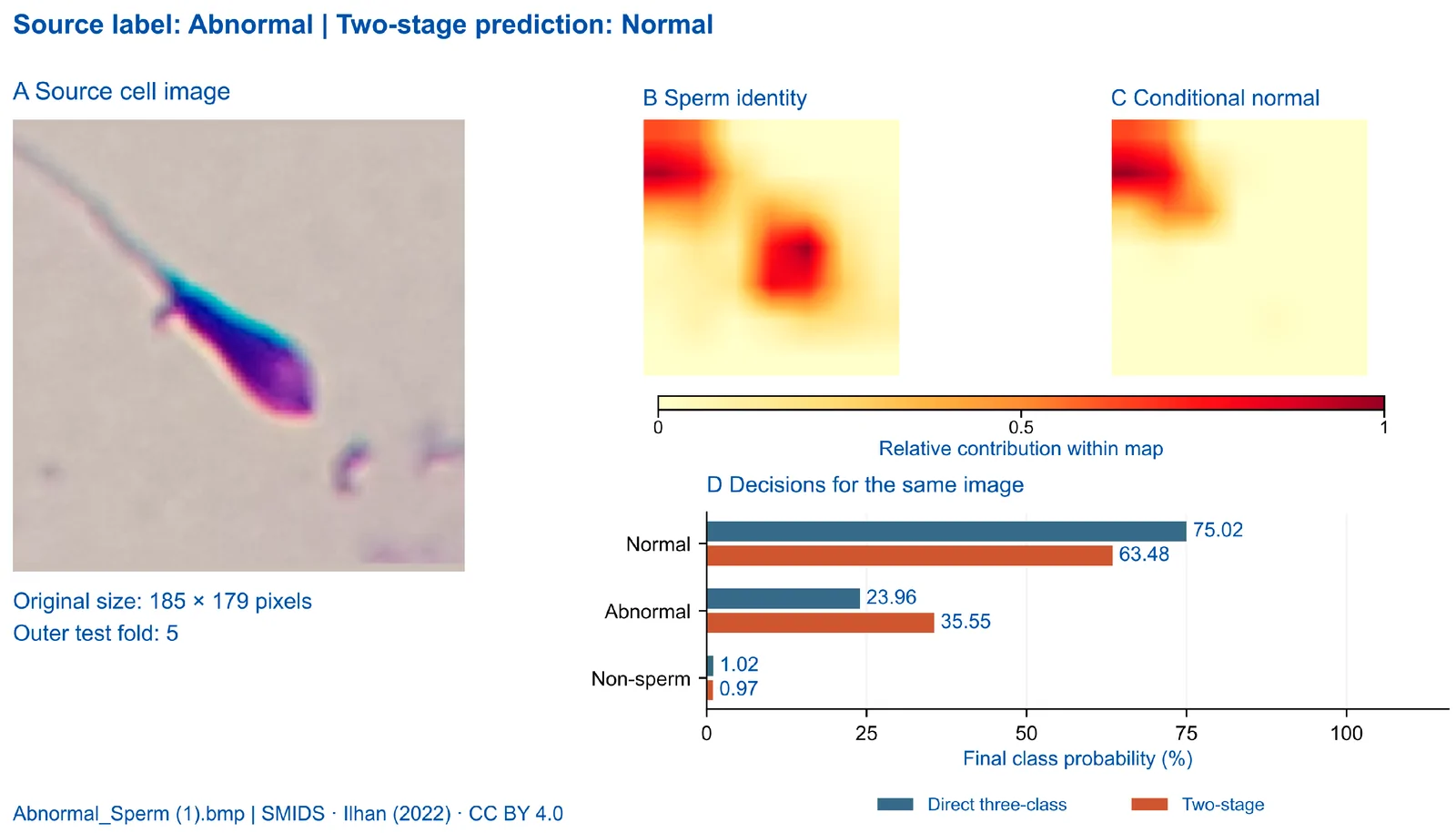
Abnormal_Sperm (1).bmp. (**A**) Source cell image padded to a square frame. (**B**) Sperm-identity and (**C**) conditional-normal Grad-CAM maps. (**D**) Final class probabilities from both models for the same image. Maps are presented in the same spatial frame as the micrograph. The color scale indicates relative contribution to the target class score and is normalized to each map’s own positive maximum. Source: SMIDS, CC BY 4.0 [25].

The third image, labeled as abnormal in the source archive, showed a tapered head profile and an elongated junction appearance. The sperm-identity probability was 99.03%; however, the conditional-normal probability was 64.10%, yielding a final normal probability of 63.48%. The direct three-class model also predicted normal with a probability of 75.02% (Figure 7). Both models would have referred this image for review at a threshold of 0.80. The combination of a high sperm-identity probability and a lower-confidence morphological decision inconsistent with the source label, at the cell level, illustrates how a two-level probability representation describes a single decision in which sperm-identity and conditional-morphology probabilities are read together; the same representation can also be derived from the direct model (Table S7). Both Grad-CAM maps for this example contain a high-contribution region in the upper-left part of the frame where the tail reaches the frame edge and in the adjacent background. The sperm-identity map also shows a high-contribution region over the head and midpiece, whereas the conditional-normal map concentrates high contributions in the upper-left region. In outer-test sperm images, the highest-contributing cell of the sperm-identity map was at the frame edge in 4.0% of images (normal 3.1%, abnormal 4.9%). For the conditional-normal map, this rate was 4.8% in normal images and 27.7% in abnormal images (nonempty maps: 928 of 972 abnormal images) (Table S1).

### 3.7. Summary of Supplementary Evaluations

Discrimination and calibration are reported in Supplementary Sections S3 and S4 and conformal prediction results in Supplementary Section S5. At the 95% conformal target, singleton rates were 65.49% for the direct model and 62.81% for the two-stage model; singleton accuracy was 94.15% and 93.84%, respectively. The direct model produced singleton outcomes for more images and had slightly higher accuracy. Additional component comparisons, matched review volumes, and class-weight sensitivity analyses are provided in Supplementary Sections S6.1–S6.3.

In unweighted retraining, overall accuracy was 84.87% for the direct model and 84.39% for the two-stage model. The two-stage model produced 85 non-sperm-to-sperm errors and 118 sperm-to-non-sperm errors. Class weights changed the magnitude of the error trade-off; overall accuracy remained higher for the direct model under both weighting conditions (Supplementary Section S6.3; Table S9).

In the evaluation using EfficientNet-B1 and EfficientNet-B2 as feature extractors, the two-stage model also showed fewer non-sperm-to-sperm errors, while the number of reference sperm images classified as non-sperm was higher. Overall accuracy was numerically highest with B1, whereas conditional-morphology accuracy of the second component was highest with B0. All Holm-adjusted *p*-values in the paired comparisons exceeded 0.05 (Tables S11 and S12).

### 3.8. Sperm-Identity Threshold Sweep and Bidirectional Errors

The sperm-identity threshold was varied using stored outer-test probabilities without retraining. The decision rule assigned an image to sperm when p(S) ≥ t and to non-sperm when p(S) < t; t varied from 0.00 to 1.00 in increments of 0.01. In the two-stage model, p(S) was the calibrated first-stage sperm-identity probability; in the direct model, it was the sum of normal and abnormal class probabilities. Both models were evaluated on the same 2947 images (1974 sperm and 973 non-sperm). Error counts were reported separately because this analysis evaluated binary identity decisions, whereas Section 3.4 evaluated three-class decisions based on the highest final class probability.

At an identity threshold of 0.50, the two-stage model erroneously accepted 83 non-sperm images and excluded 123 reference sperm images; the corresponding counts for the direct model were 106 and 69. Identity area under the receiver operating characteristic curve (ROC-AUC) values were 0.978 and 0.979 for the two-stage and direct models, respectively. At these similar levels of discrimination, the threshold sweep showed how the decision threshold changed the distribution of bidirectional errors. Descriptive thresholds based on Youden’s J statistic and the proximity of the two error rates are reported in Supplementary Section S2.4.

**Figure 8:**
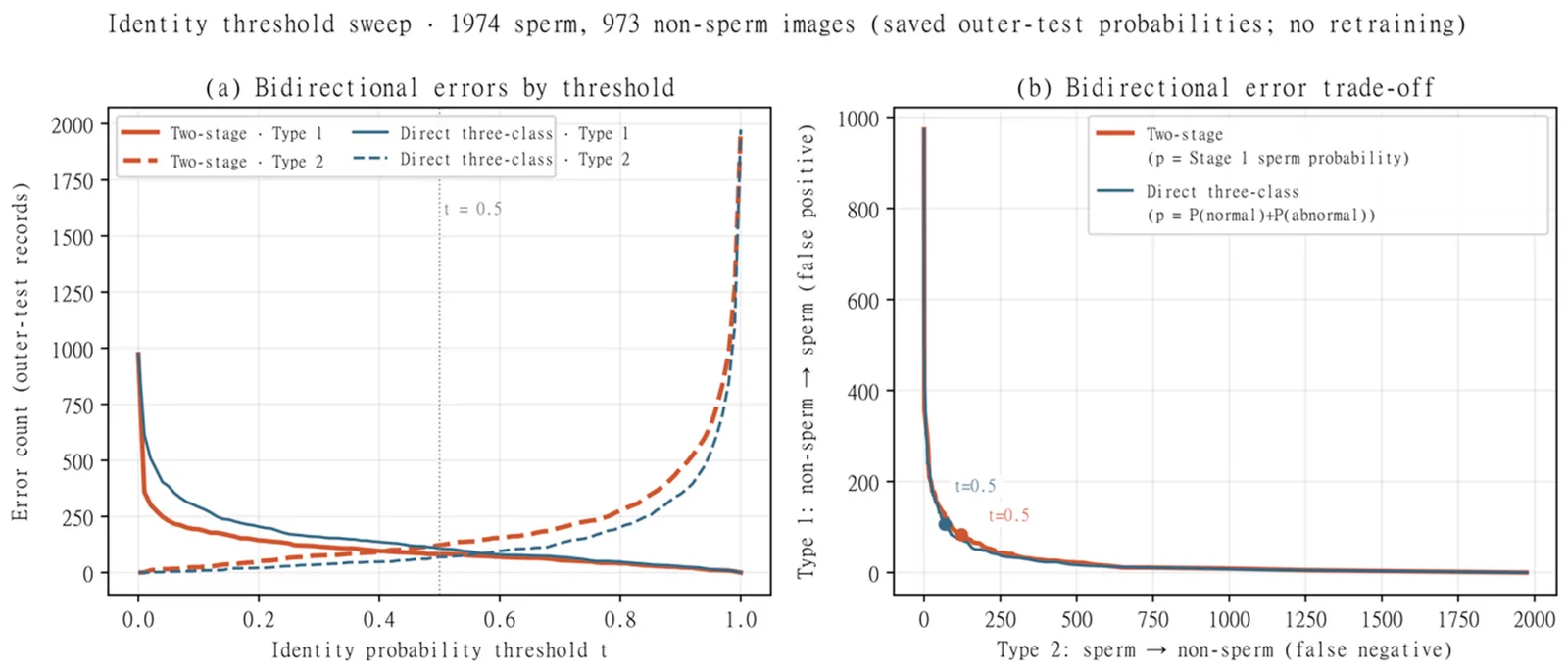
Relationship between the sperm-identity probability threshold and bidirectional error counts. (**a**) Erroneous acceptance of non-sperm images (Type 1) and exclusion of reference sperm images (Type 2) as the threshold varies from 0.00 to 1.00. (**b**) Trade-off between the two error directions; marked points indicate the 0.50 identity threshold. Calculations used stored outer-test probabilities without retraining. Sperm-identity probability was obtained from the calibrated first-stage output in the two-stage model and from the sum of normal and abnormal probabilities in the direct model. These binary threshold decisions differ from the three-class decisions based on the highest final class probability in Figure 3.

In the two-stage model, lowering the identity threshold from 0.50 to 0.40 reduced reference sperm exclusions from 123 to 93 while increasing erroneous acceptance of non-sperm images from 83 to 96 (Figure 8). Thus, the increase in erroneous acceptance accompanying preservation of assessable sperm images was quantified on the same records. Error counts and class-specific error rates at selected thresholds for both models are provided in Supplementary Section S2.4 and Table S4.

## 4. Discussion

The two-stage and direct models showed different distributions of sperm-identity errors using shared image features and the same outer-test records. Misclassification of non-sperm images as sperm decreased from 94 (direct model) to 77 (two-stage model), whereas misclassification of reference sperm images as non-sperm increased from 82 to 141. This difference in error direction reflects different operating points of two models with similar sperm-identity discrimination. From a histomorphological perspective, this finding shows that exclusion of non-sperm material and preservation of cells suitable for morphological assessment should be monitored together. Cell identification and interpretation of normal–abnormal appearance are distinct assessment tasks [3,5]. The contribution of this study is to show how decisions on the same images differ between the separate and direct learning of these targets while accounting for review volume.

The SMIDS source study reported 87% accuracy for three-class classification using MobileNet [20]. Other studies reported 92.5% accuracy with BEiT-Base [21] and 96.08% accuracy with CBAM-enhanced ResNet50 features combined with a support vector machine [22]. These publications examine the performance of different image representations and classifiers. In the main analysis, frozen EfficientNet-B0 features provided shared input for the learning structures being compared. The sperm-identity and conditional-morphology components were thus evaluated separately from representation changes. While our two models were compared directly on the same test records, percentages from previous publications were considered within their methodological contexts because preprocessing and validation conditions differed [31]. Dataset targets, methods, and reported results are compared in Supplementary Section S6.4. Evaluating pretraining and feature transfer in medical imaging in relation to task and model capacity further supports this distinction [32]. Yang et al. [33] reported 90.82% morphological accuracy using EfficientNet-B0 in a system integrating live sperm tracking and regional segmentation. That value describes the entire system and concerns a task different from the normal, abnormal, and non-sperm classification of static SMIDS images evaluated here.

Overall accuracy was 84.80% for the direct model and 83.98% for the two-stage model; selective accuracy at matched review volumes was also higher for the direct model. These results indicate that the two learning structures should be compared in terms of error direction as well as overall performance. A preliminary classifier intended to reduce erroneous acceptance of non-sperm material should also monitor the exclusion of true sperm. The relative importance of these two errors in the target workflow is central to implementation choice. Variation in sperm detection performance across laboratory conditions [31] and the fact that archive class proportions do not represent ejaculate morphology [5,14] require consideration of the target laboratory’s imaging conditions and cell distribution before deployment.

The anatomical scope of a dataset determines the question a model answers. SCIAN-MorphoSpermGS provides a reference for sperm head shapes, whereas Hi-LabSpermMorpho covers head, neck, and tail appearances in 18 expert-labeled classes [15,17]. Studies addressing different morphological targets support separate definitions of regional visibility and defect labels [18]. Shahali et al. [4] evaluated whole-cell morphology in live, unstained sperm using expert consensus. Turkoglu et al. [24] reported accuracies of 69.43%, 71.34%, and 68.41% under three staining conditions; their anatomically grouped two-stage approach targeted 18 morphology classes. Here, the first component learns sperm identity, and the second learns normal–abnormal classification conditional on sperm identity. This distinction reflects the aim of two-stage designs to express different histomorphological questions as separate decision levels; the same two levels can also be derived from the direct model. Other datasets provide comparative context; only SMIDS was used for training.

The three purposively selected cells illustrate normal and abnormal decisions consistent with source labels and an erroneous normal decision within a shared visual layout (Figure 5, Figure 6 and Figure 7). Presenting the source micrograph, stage-specific Grad-CAM maps, and final class probabilities together allows cell appearance to be compared with the model response. Grad-CAM maps show the spatial distribution of relative contributions to the class score [28]. Histomorphological interpretation describes the visible head and junction characteristics of the selected cells and their relationship to the source label and model decision. Associations between genetic causes and morphological defects [34] and between residual cytoplasm and oxidative stress [35] explain the biological significance of morphology. Joint evaluation of structural image characteristics and functional measurements is a research direction for linking morphological classification to its biological correlates.

The duplicate-image audit and retention of similarity groups within the same validation fold were methodological safeguards against repetition of image content between training and test data. Retaining record identifiers, group assignments, and outer-test probabilities also allowed each image’s model decision and experimental role to be traced. Because a model may use background cues as well as anatomical structures for classification [36], staining, illumination, and background effects should be examined further using images from different sources [31]. Grad-CAM maps accompany three purposively selected test images as descriptive examples; the frame-edge analysis covers all outer-test images for the sperm-identity map and all reference sperm images for the conditional-normal map (Table S1). In the misclassified example in Figure 7, both maps indicated a high-contribution region in the upper-left part of the frame. In sperm images, the highest-contributing cell of the sperm-identity map rarely lies at the frame edge; for the conditional-normal map, this occurs mainly in abnormal images. This pattern may reflect background or acquisition-related cues of the kind described as shortcut learning [36]. At the 7 × 7 resolution of the final convolutional layer (224 × 224 input), an edge cell includes both the tail tip and the adjacent background. Distinguishing tail from background contributions requires region-level annotations beyond the image-level labels available in SMIDS.

The contribution of a histology and embryology specialist extends beyond assigning reference labels to defining morphological criteria, visibility limits, and interobserver disagreements [37]. The consensus approach of Shahali et al. [4] provides an example based on independent classifications by three andrology specialists. Separate classes for anatomical defects and interobserver variability in morphological criteria emphasize the importance of regional records [15,38]. These findings support separate recording of head, acrosome, neck–midpiece, and tail characteristics in future studies examining the anatomical basis of model decisions. In this study design, in which source labels were retained, expert input concerned definition of assessment targets and cellular interpretation. The quantitative effect of expert involvement can be compared under expert-only, model-only, and combined-use conditions [39].

Model probabilities quantify confidence in assigning an image to each source class. Discrimination and probability calibration were therefore reported separately [27,40]. The matched review volume analysis compared remaining errors when each model retained decisions for the same number of images. The direct model achieved higher selective accuracy by this criterion; two-stage results were considered alongside changes in identity-error directions. At the 95% conformal target, the singleton rate and singleton accuracy were also higher for the direct model. Conformal prediction reports possible classes as a set and assumes exchangeability between calibration and new observations [29,41]. Because the approximately balanced archive distribution differs from routine microscopy, precision, calibration, and expected review volume should be reassessed in the target laboratory [42].

The identity-threshold sweep showed how the relationship between erroneous acceptance of non-sperm material and exclusion of reference sperm images changes with the decision threshold (Figure 8). The threshold was applied to the calibrated first-stage probability in the two-stage model and to the sum of normal and abnormal probabilities in the direct model. The effect of threshold variation was therefore evaluated on the same images for both learning structures. Lowering the threshold in the two-stage model preserved more reference sperm images for assessment while also accepting more non-sperm images. Given the similar levels of sperm-identity discrimination, the main contribution of this threshold comparison is the joint quantification of how threshold selection affects both error directions. The direct model retains its small overall accuracy advantage in the main three-class comparison. In a workflow that prioritizes exclusion of non-sperm material, the accompanying loss of true sperm images and the possibility of expert reassessment are also part of model selection. This evaluation provides a framework for considering AI-assisted semen analysis and sperm selection in their application context [43,44]. The clinical relevance of this framework is determined by evaluating its contribution to the expert workflow within the relevant task and conditions of use [45].

The intended application is preliminary classification of acquired microscopy image patches, with selected images referred for expert review. The histological and embryological perspective distinguishes sperm-identity errors from morphology errors and relates visible anatomical characteristics to model decisions. Expert reassessment, acquisition of additional images when necessary, and recording of the final decision are components of prospective workflow evaluation [39,46]. Laboratory quality assurance and clinical interpretation remain the responsibility of trained specialists and the relevant clinical team [10].

Morphological classification and sperm selection for treatment are separate applications. Intrauterine insemination (IUI) uses a prepared population of motile sperm, whereas injection of a single spermatozoon into the oocyte is specific to intracytoplasmic sperm injection (ICSI) [5,10]. The meta-analysis by Kohn et al. [47] supports not restricting IUI options solely on the basis of morphology under appropriate clinical conditions. Findings should be interpreted alongside other semen characteristics and the couple’s clinical data [8]. Extending assessment from static SMIDS images to live-cell selection requires separate validation for motility, vitality, and appropriate imaging conditions [4].

The effect of AI assistance on expert workflows has been examined in different sperm assessment tasks. Goss et al. [45] reported reduced embryologist review time with AI assistance in a simulation of clinical rare-sperm searching. However, model computation time differs from total workflow time, which includes sample preparation and reporting [4,39]. The increasing population-level burden of infertility supports the need to expand service capacity [48]. The numbers of review candidates identified here may inform prospective studies of expert workload. Such evaluations should treat image count, expert review time, and cost as separate endpoints.

In a binary normal–abnormal evaluation involving SMIDS, HuSHeM, and Hi-LabSpermMorpho datasets, AlSaad et al. [23] reported a source-balanced mean macro-F1 of 0.91 for fully fine-tuned SigLIP2 and a macro-F1 of 0.89 in the SMIDS subset. These results evaluate representation adaptation and cross-source performance. The present study focuses on how cell acceptance and exclusion decisions differ between separate and direct learning of identity and morphology on shared test records that also include the non-sperm class. Preliminary histomorphological assessment was therefore considered alongside error direction and decisions at matched review volume (Supplementary Section S6.4).

In supplementary comparisons using B1 and B2, changes in bidirectional identity errors followed the same direction as in the B0 experiment. Non-sperm-to-sperm errors decreased from 94 to 77 with B0, from 84 to 56 with B1, and from 92 to 61 with B2; conversely, misclassification of true sperm as non-sperm increased with all three feature extractors. These findings describe the error distributions of the two learning structures under the frozen representations examined. Numerical differences between feature extractors were reported with confidence intervals and Holm-adjusted *p*-values. This comparison evaluates sensitivity to feature-extractor choice; fine-tuning and transfer across laboratories can be investigated in separate experimental designs (Supplementary Section S6.6).

## 5. Study Scope and Limitations

The unit of evaluation was the image defined by its SMIDS source label. Because the archive lacks patient and slide identifiers, possible shared-source dependence between similarity groups should be considered when interpreting the statistics. Bootstrap confidence intervals and the McNemar test describe image-level comparisons, whereas conformal results express empirical coverage under the available group structure. Cellular panels are descriptive examples; without regional defect labels or new expert assessment, they do not constitute anatomical validation or defect segmentation. Distinguishing tail and background contributions at the frame edge using region-level annotated data is the immediate next step. The evaluated endpoints were classification, error direction, and review candidacy. Expert correction success, time, cost, and clinical outcomes were not measured in this experiment. The proportion of normal forms in an ejaculate, infertility diagnosis, and live sperm selection are separate clinical assessment targets. Image dimensions differ across classes (abnormal images are larger and more variable; median width 176 px versus 164 px for normal); this information may reach the model after square padding. Translation into practice entails independent validation across different patients, slides, and laboratories and prospective evaluation of the expert–model workflow.

## 6. Conclusions

The two-stage and direct models differed in the trade-off between erroneous acceptance of non-sperm material and exclusion of true sperm images under shared image features and test partitions. The two-stage model showed fewer non-sperm-to-sperm misclassifications and more exclusions of true sperm than the direct model; overall accuracy and selective accuracy at matched review volume were higher for the direct model. This comparison indicates that correct cell identification and preservation of assessable sperm cells should be monitored together in preliminary histomorphological assessment.

The contribution to the literature is a paired analysis of decisions on the same test images that differ between conditional and direct learning, interpreted through bidirectional identity errors and review volume. Cellular panels allow these decisions to be examined alongside visible anatomical characteristics. This comparison supports evaluation of models for andrology laboratories in terms of erroneously accepted material, excluded sperm images, and errors remaining outside review, as well as overall accuracy. These criteria may contribute to prospective evaluation of expert-assisted workflows.

The supplementary identity-threshold analysis showed that erroneous acceptance and sperm exclusion counts changed with threshold selection in both models. This finding supports evaluating the trade-off between preserving assessable cells and separating non-sperm material by considering model structure and the decision rule together.

## Figures and Tables

**Table 1:** Image distribution in the Sperm Morphology Image Dataset (SMIDS) before and after the consistency audit.

Data Stage	Normal	Abnormal	Non-Sperm	Total
Original images	1021	1005	974	3000
Included images	1002	972	973	2947
Excluded images	19	33	1	53

Counts refer to image files. Source labels were unchanged; class proportions do not represent the morphological composition of an ejaculate.

**Table 2:** Shared image representation and classification components. CNN: convolutional neural network; L-BFGS: limited-memory Broyden–Fletcher–Goldfarb–Shanno algorithm.

Component	Task	Implementation
EfficientNet-B0	Image feature extraction	Frozen CNN pretrained on ImageNet; 1280 features.
Direct three-class model	Joint prediction of normal, abnormal, and non-sperm classes	StandardScaler + three-class logistic regression; L-BFGS solver.
Stage 1	Binary classification of sperm identity	StandardScaler + binary logistic regression; liblinear; sperm as the positive class.
Stage 2	Binary classification of abnormality within sperm images	StandardScaler + binary logistic regression; liblinear; abnormal as the positive class.

Stages 1 and 2 form the proposed model; the direct three-class model is the comparator. Gradient-weighted class activation mapping (Grad-CAM) was used for post hoc explanation rather than as an additional classifier.

**Table 3:** Final classification performance in pooled outer-test predictions (n = 2947). MCC: Matthews correlation coefficient; ECE: expected calibration error; log loss: logarithmic loss. Macro-F1 is the equally weighted mean of class-specific F1 scores.

Metric	Direct Three-Class Model	Two-Stage Model
Correct/incorrect image count	2499/448	2475/472
Accuracy	84.80%	83.98%
Balanced accuracy	84.81%	84.00%
Macro-F1	0.848	0.839
MCC	0.772	0.760
Log loss	0.398	0.416
Multiclass Brier score	0.223	0.233
Ten-bin ECE	0.015	0.019

**Table 4:** Separate performance of the sperm identity and morphology components. MCC: Matthews correlation coefficient; F1: harmonic mean of precision and sensitivity.

Metric	Stage 1	Stage 2
Images evaluated	2947	1974
Positive class	Sperm	Abnormal
Accuracy	93.01%	85.46%
Balanced accuracy	92.62%	85.45%
Positive-class sensitivity	93.77%	84.47%
Negative-class specificity	91.47%	86.43%
Positive predictive value	95.71%	85.79%
F1	0.947	0.851
MCC	0.844	0.709

**Table 5:** Class-specific performance in pooled outer-test predictions.

Model	Class	n	Sensitivity	Precision	F1
Direct three-class	Normal	1002	83.23%	83.40%	0.833
Direct three-class	Abnormal	972	80.86%	79.72%	0.803
Direct three-class	Non-sperm	973	90.34%	91.47%	0.909
Two-stage	Normal	1002	82.34%	84.01%	0.832
Two-stage	Abnormal	972	77.57%	81.25%	0.794
Two-stage	Non-sperm	973	92.09%	86.40%	0.892

Sensitivity is the proportion of images in a reference class that are correctly identified; precision is the proportion of predictions for that class that agree with the source label. n denotes the number of reference images: 1002 normal, 972 abnormal, and 973 non-sperm images.

## Data Availability

SMIDS version 1 is openly available on Mendeley Data under the Creative Commons Attribution 4.0 license: https://doi.org/10.17632/6xvdhc9fyb.1 [25]. Source images and labels were obtained from this archive. The analysis reproducibility package includes identifiers of included images, relative source paths, similarity groups, outer-fold assignments, stored probabilities, and reproduction code. Source images are not redistributed in the package. The reproducibility package is deposited on Zenodo: https://doi.org/10.5281/zenodo.23211652 [49]. The code in the package is released under the MIT License. SMIDS images are licensed under CC BY 4.0 and are accessible from the source record [25]. ImageNet-pretrained EfficientNet weights are subject to their providers’ terms.

## Supplementary Materials

The following supporting information can be downloaded at: https://www.mdpi.com/article/10.3390/diagnostics16203390/s1, Section S1 presents detailed methods, Section S2 outer-test results, Section S3 discrimination, Section S4 calibration, Section S5 the conformal experiment, and Section S6 supplementary comparisons. Figures S1–S4 show discrimination, calibration, and conformal results; Figure S5 shows selective accuracy at matched coverage. Table S1 presents the location of the highest Grad-CAM contribution in outer-test images; Tables S2 and S3 present outer-fold and bootstrap results; Table S4 presents bidirectional error counts at selected sperm-identity thresholds; Tables S5 and S6 present conformal results; Table S7 presents the derived-component comparison; Table S8 presents matched review volume; Table S9 presents class-weight sensitivity; Table S10 presents the literature comparison; and Tables S11 and S12 present the supplementary B1–B2 evaluation and paired accuracy comparisons between feature extractors. The reproducibility package contains image identifiers, similarity groups, fold assignments, predictions, and analysis code.

## Abbreviations

The following abbreviations are used in this manuscript:

AI	Artificial intelligence
BMP	Bitmap
CBAM	Convolutional block attention module
CNN	Convolutional neural network
ECE	Expected calibration error
ESHRE	European Society of Human Reproduction and Embryology
Grad-CAM	Gradient-weighted class activation mapping
ICSI	Intracytoplasmic sperm injection
IUI	Intrauterine insemination
IVF	In vitro fertilization
L-BFGS	Limited-memory Broyden–Fletcher–Goldfarb–Shanno
MCC	Matthews correlation coefficient
RGB	Red–green–blue
ROC-AUC	Area under the receiver operating characteristic curve
SMIDS	Sperm Morphology Image Dataset
WHO	World Health Organization
