# Supplementary material for: Two-Stage Probabilistic Classification of Sperm Identity and Morphology
Source: Diagnostics (Basel). 2026 Oct 18;16(20):3390. doi: 10.3390/diagnostics16203390 (PMC13703208; doi:10.3390/diagnostics16203390)
Supplement: Supplementary file 1 [file diagnostics-16-03390-s001.zip › diagnostics-4604969-supplementary.pdf]

Supplementary Material

Two-Stage Probabilistic Classification of Sperm Identity and Morphology

S1. Supplementary Methods

S1.1. Principles of Histomorphological Assessment

Visual descriptions based on World Health Organization (WHO) criteria were restricted to the head contour, head–tail junction, and tail segment discernible in each image. Light and dark regions within the head and proximal thickening were described as image characteristics; no new labels were generated for acrosomal, nuclear, or mitochondrial defects. General normal–abnormal labels were retained, and the anatomical diagram provided an explanatory framework independent of the micrographs [5].

Source images in the Sperm Morphology Image Data Set (SMIDS) are bitmap (BMP) files with red–green–blue (RGB) color channels. Staining characteristics, texture, and cell shape were unchanged. Arrows indicated only visible structures. Because physical calibration was unavailable, no micrometer scale bar was generated; different fields of view were not used to compare cell size. Schematic drawings were not added to training or test data. Original filenames ensured traceability within the archive [25].

S1.2. Image Selection and Cellular Contribution Maps

For gradient-weighted class activation mapping (Grad-CAM), a pool of 24 images comprising the first 12 normal and first 12 abnormal records in the fifth outer-test fold was used. One normal image, one abnormal image, and one abnormal image misclassified as normal were purposively selected to illustrate visible anatomy and different model decisions. This selection exemplified cellular decisions; overall performance was calculated from all outer-test images.

Grad-CAM was calculated from the 1280-channel, 7 × 7 activations of the final EfficientNet-B0 block with respect to the class score, including feature standardization, logistic regression, and temperature calibration [28]. The sperm logit was targeted in the first component, and the predicted normal or abnormal logit in the second. A rectified linear unit (ReLU) was applied to activations weighted by spatially averaged gradients; each map was divided by its own positive maximum. Stored maps were resized to 224 × 224 pixels by bilinear interpolation and displayed on a yellow–red scale without further value modification. The source micrograph appeared on the left, smaller stage-specific maps at the upper right, and final class probabilities at the lower right. Median-color padding, preserving aspect ratio of the cell image, produced a square frame. Source dimensions were indicated in the figures. Maps were calculated at a spatial resolution of 7 × 7 and displayed at 224 × 224 pixels. Source pixels, probabilities, and selected examples were unchanged. Maps show relative spatial contributions to the target class score of the respective component; combined final class probabilities are presented in a separate plot. To describe how common this pattern was, we calculated two maps across the outer-test images: a sperm-identity map (Stage 1, sperm target) for all 2947 images and a conditional-normal map (Stage 2, normal target) for the 1974 reference sperm images. For each map, we recorded whether the highest-contributing cell was at the frame edge of the 7 × 7 grid (24 of 49 cells), whether it was at a corner (4 of 49 cells), and the share of total contribution carried by the frame edge. Zero-contribution maps were excluded from denominators; 95% Wilson confidence intervals are provided (Table S1-). Maps follow the Grad-CAM formulation in training/single_cell_gradcam.py in the reproducibility package, with the same score definition (calibrated stage logit and temperature) [49].

Table S1-. Location of the highest Grad-CAM contribution in outer-test images. CI, confidence interval; IQR, interquartile range. The sperm-identity map shows the sperm target in Stage 1; the conditional-normal map shows the normal target in Stage 2 and covers 1974 reference sperm images.

Map and true class	n	Empty maps	Peak cell at frame edge, % (95% CI)	Peak cell at corner, %	Frame-edge contribution share, median (IQR)
Sperm identity, Normal	1002	0	3.1 (2.2–4.4)	0.2	0.157 (0.104–0.217)
Sperm identity, Abnormal	972	0	4.9 (3.7–6.5)	0.3	0.162 (0.101–0.224)
Sperm identity, Non-sperm	973	115	45.5 (42.2–48.8)	10.4	0.388 (0.089–0.732)
Conditional normal, Normal	1002	0	4.8 (3.6–6.3)	0.6	0.079 (0.019–0.182)
Conditional normal, Abnormal	972	44	27.7 (24.9–30.7)	6.6	0.243 (0.035–0.521)

The frame edge covers 24 of 49 cells (48.98%); zero-contribution maps are excluded from percentages.

S1.3. Decision Transitions and Probability Plots

Direct and two-stage decisions were compared by pairing outer-test predictions for each image by record identifier. Receiver operating characteristic (ROC) and precision–recall (PR) curves were generated from pooled outer-test probabilities. The area under the ROC curve (AUC) was termed ROC-AUC. A one-versus-rest approach was used for the three final classes. The positive class was sperm in Stage 1 and abnormal in Stage 2, the latter evaluated only within reference sperm images. PR performance was summarized by average precision (AP), calculated as precision weighted by increments in recall; it was not treated as equivalent to trapezoidal PR area.

The two-stage structure is a decision framework that jointly evaluates sperm-identity and conditional-morphology probabilities. Stage 1 produces the sperm-identity probability; Stage 2 learns the normal–abnormal probability conditional on sperm identity using only sperm-labeled development images. At test time, features from the same image are supplied to both components, and their outputs are converted into composite probabilities. The sum of final normal and abnormal probabilities equals the sperm-identity probability. When this probability is below 0.50, the final non-sperm probability exceeds the final probability of either morphological class.

Calibration plots compared mean probability with observed frequency in ten equal-width bins spanning 0–1. For final decision confidence, the observed frequency was accuracy; in class-specific plots, it was the proportion belonging to the respective source class. Plots described probability agreement in previously generated model predictions. Confidence–coverage results were recorded as outputs of the main experiment; conformal results came from a supplementary experiment retrained with a separate calibration partition.

S1.4. Split-Conformal Classification

The five outer-test folds of the main experiment were unchanged. Within each outer-training set, the number of groups reserved for conformal calibration was determined by rounding 20% of the similarity groups up to the nearest integer. Groups were randomly selected without class stratification. Standardization, selection of the inverse regularization parameter (C) using three inner folds, and temperature calibration were repeated on the remaining development data. Conformal calibration images were excluded from these steps. Both models used the same development, calibration, and test partitions; Stage 2 was trained only on reference sperm images in the development set.

The supplementary conformal experiment also applied no hard acceptance or rejection decision at Stage 1. The sperm identity probability from Stage 1 and the conditional-morphology probability from Stage 2 were used jointly; conformal prediction sets were constructed from the combined final probabilities.

The nonconformity score was defined as one minus the final probability assigned to the source class. To avoid treating similar images as independent calibration observations, the maximum score within each similarity group was used. For m calibration groups, the threshold was the ordered score at rank $(m + 1) \times (1 - \alpha)$, rounded upward. Classes whose scores did not exceed this threshold were included in the prediction set for a new image. The target error level was denoted by α; values of 0.05 and 0.10 were prespecified for target coverage of 95% and 90%, respectively [29].

The proportion of sets containing the source label, singleton rate, accuracy within the singleton subset, and referral rate for non-singleton results were calculated. Group-level coverage, defined as the proportion of groups in which every image's source label was included in its own prediction set, and class-specific coverage were also reported. Singleton results were considered candidates for automated assessment, and other results candidates for research-level expert review; this distinction was not applied to clinical reporting.

The finite-sample coverage guarantee assumes exchangeability between calibration and new observation units [29]. The unit in this experiment was the image-similarity group; because patient and slide identifiers were unavailable, no patient-level coverage guarantee was provided. Values pooled across the five outer folds were reported as empirical coverage. Point estimates from this arm, which used fewer development images because of separate calibration, were not combined with the main-experiment results.

S1.5. Validation and Performance Metrics

Five outer and three inner folds were generated using StratifiedGroupKFold. Random seeds were 20260915 for outer folds and 20260915 + 100 × outer-fold number for inner folds. For each candidate C (0.01, 0.1, and 1.0), StandardScaler and logistic regression were fitted only on the inner-training partition. Inner-validation predictions were pooled to calculate balanced accuracy; ties favored the smaller C. Temperature was selected using out-of-fold probabilities for the chosen C by minimizing log loss over 0.25–4.0 through bounded one-dimensional minimization [27]. C selection and temperature selection used the same inner-fold predictions and were treated as two steps of a shared inner model-development process. The selected model was refitted using all outer-training data; the second component was refitted on the reference sperm subset. Outer-test images were excluded from these selections. The frozen ImageNet feature extractor was not retrained on this archive. Thus, inner folds were used for model development, whereas performance was calculated from outer-test predictions held separate from selection procedures. Parameters learned from the archive data belonged to standardization, logistic regression, and temperature calibration. The pretrained representation provided shared input to both learning structures; task-specific interpretation of transfer in medical imaging has been discussed by Raghu et al. [32].

Classification performance was evaluated using accuracy, balanced accuracy, class sensitivity, precision, F1 score, macro-F1, and the Matthews correlation coefficient (MCC). Balanced accuracy and macro-F1 were calculated as equally weighted means of class sensitivities and class-specific F1 scores, respectively. Discrimination was examined using receiver operating characteristic (ROC) and precision–recall (PR) curves. Area under the ROC curve (AUC) was calculated for each stage and the three final classes using a one-versus-rest approach and reported as ROC-AUC. Agreement between predicted probabilities and observed outcomes was evaluated using log loss, the multiclass Brier score, ten-bin expected calibration error (ECE), and calibration plots.

Decisions on the same test images were paired by record identifier. Using fixed outer-fold predictions, similarity groups were sampled with replacement 2000 times to calculate 95% bootstrap confidence intervals; models were not retrained in each iteration. The two-sided exact McNemar test was applied to paired correct–incorrect decisions. Statistical interpretation was based on the available image-similarity groups. The absence of patient and slide identifiers limits further adjustment for shared-source dependence between groups; confidence intervals and the p-value were interpreted within this image-level scope.

S1.6. Software

Analyses were performed in Python 3.12 using PyTorch 2.13.0 and torchvision 0.28.0 for deep feature extraction and Grad-CAM visualization, and scikit-learn 1.9.0 for statistical learning and model evaluation. Image processing, numerical analyses, and plotting were supported by standard Python libraries.

Generative AI assistance. The use of OpenAI ChatGPT and Codex is described in the Materials and Methods section of the main manuscript. The same disclosure applies to this supplementary material. Illustrative schematics did not replace source micrographs, source class labels, or training and test data. The authors reviewed and edited the generated content and take responsibility for the scientific accuracy of the text, code, results, and interpretations.

S1.7. Duplicate-Record Audit and Image Groups

To identify pixel-identical images, each image was converted to RGB, and its width, height, and pixel bytes were included in a SHA-256 hash. For identical images with the same label, the first record in file order was retained and the others removed. When a hash corresponded to multiple source labels, all records were excluded. This removed 45 redundant same-label copies and eight files in four conflicting-label pairs. For near-duplicate screening, images were resized to 32 × 32 grayscale pixels, and 64-bit perceptual hashes were generated from the upper-left 8 × 8 discrete cosine transform coefficients; the direct-current coefficient was excluded when calculating the median threshold. The 1125 candidate pairs with a Hamming distance of at most 2 were compared using the structural similarity index (SSIM) on aspect-ratio-preserving 64 × 64 grayscale images. Five pairs meeting SSIM $\geq$ 0.995 were assigned to shared connected components. This yielded 2942 groups; five contained two images, and none contained mixed labels. Groups were defined by computational thresholds. Candidate pairs did not undergo independent expert visual verification; the grouping unit was computational image similarity. Identifiers and outer-fold assignments are included in the package described in S6.5.

S1.8. Supplementary Comparisons

Supplementary analyses planned after the main experiment used stored outer-test predictions. Sperm-identity probability was derived from the direct model as p_d(N)+p_d(A), and conditional-abnormal probability as p_d(A)/[p_d(N)+p_d(A)], with numerical protection against a zero denominator. Components were compared within the same tasks, using all 2947 images for sperm identity and 1974 reference sperm images for conditional morphology. The threshold was 0.50. In the matched-review-volume analysis, confidence was the maximum of each model's three final class probabilities. Images were ranked by decreasing confidence; ties were resolved by increasing record identifier. The same number of high-confidence decisions was retained, and remaining images were counted as review candidates. Coverage levels of 50%, 60%, 70%, 80%, and 90%, together with the original retained-decision counts of 1974 and 2022 at the 0.80 threshold, were evaluated. Thresholds described the relationship between confidence and coverage in stored predictions. For class-weight sensitivity, models were retrained with class_weight=None while retaining the same features, outer folds, candidate C values, inner selection, and temperature calibration. The balanced condition was also retrained to verify reproduction of the original probabilities. Results are provided in S6.1–S6.3. The 0.80 threshold is an operating point used to examine retained decisions and review-candidate counts together. Clinical threshold selection requires prospective evaluation under target laboratory conditions. Confidence and coverage were considered within the selective classification framework [30]. Stored predictions were described on a fixed threshold grid; the guaranteed-risk selection algorithm from that publication was not applied. Matched review volume was used to compare models on the same number of retained decisions, rather than as a separate mechanism for generating uncertainty.

S2. Outer-Test Results and Decision Comparisons

S2.1. Outer Folds and Resampling

Each image was evaluated exactly once in an outer-test set. The first two test folds contained 590 images each, and the remaining three contained 589 each. Corresponding outer-training sets contained 2357 or 2358 images. Each outer-training set contained approximately 1578–1580 reference sperm images for Stage 2. The direct three-class model achieved slightly higher balanced accuracy than the two-stage model in all five folds. Fold sizes and model settings are provided in Table S1; S1 denotes the sperm-identity component, S1 the morphology component, and n the number of test images.

Table S2. Outer-test fold sizes and fold-level results. n, number of images; C, inverse regularization parameter; S1, sperm-identity component; S2, conditional-morphology component.

Fold	Test n	Normal / abnormal / non-sperm	Direct balanced accuracy	Two-stage balanced accuracy	Selected C (direct / S1 / S2)
1	590	201/195/194	83.11%	81.75%	0.01/0.01/0.01
2	590	200/195/195	83.02%	82.87%	0.01/0.10/0.01
3	589	200/194/195	87.80%	87.64%	0.01/0.10/0.01
4	589	201/194/194	85.60%	84.57%	0.01/0.10/0.01
5	589	200/194/195	84.54%	83.16%	0.01/0.01/0.01

C is the inverse of regularization strength in logistic regression; smaller values indicate stronger regularization. Values were selected by inner cross-validation and are listed in the order of the direct three-class model, sperm-identity component (S1), and conditional-morphology component (S2). The grouping unit for fold construction was the image-similarity group.

C = 0.01 was selected in every outer fold for the direct model and Stage 2. For Stage 1, C = 0.01 was selected in two folds and C = 0.1 in three; the selected values favored strong regularization.

Selected temperatures ranged from 1.123 to 1.231 for the direct model, from 0.905 to 1.510 for Stage 1, and from 1.092 to 1.287 for Stage 2.

The 95% confidence interval for the balanced accuracy difference remained below zero (Table S3). The image-level exact McNemar test yielded $p = 0.0369$; 73 images were classified correctly only by the direct model and 49 only by the two-stage model. This difference concerns the comparison under the image-group-based validation and resampling conditions described in Methods.

Table S3. Paired bootstrap results for balanced accuracy.

Metric	Point estimate	95% confidence interval
Direct balanced accuracy	84.81%	83.54%–86.08%
Two-stage balanced accuracy	84.00%	82.74%–85.25%
Difference, two-stage minus direct	−0.81 percentage points	−1.55 to −0.08 percentage points

Results are based on 2000 group-bootstrap iterations. The difference was calculated as two-stage minus direct. Intervals were calculated from fixed outer-fold predictions.

S2.2. Paired Decision Transitions

Record-level pairing showed that the models disagreed on 136 images (4.61%). Only the direct model was correct in 73 cases, and only the two-stage model in 49; in 14 images, the models selected different classes but both were wrong. Twenty-one normal images classified as abnormal by the direct three-class model were classified as normal by the two-stage model. Conversely, eight images correctly classified as normal by the direct model were classified as abnormal by the two-stage model; 111 erroneous abnormal decisions were shared. Twelve source non-sperm images classified as abnormal by the direct model were correctly excluded by the two-stage model. These counts describe both corrected and misclassified images after the transition to two-component classification.

In the stored probabilities, 24 images had a Stage 1 sperm identity probability of at least 0.50 but were classified as non-sperm in the combined three-class decision. These records show that probabilistic combination determines the final class through the joint contribution of both components. The probability from the first component determines the final class together with the second component's uncertainty between normal and abnormal. True sperm excluded by a hard-threshold system can also be counted against source labels. In the present comparison, retaining or deriving component probabilities allows joint examination of each image's identity and conditional-morphology responses (Section S6.1; Table S7).

S2.3. Confidence-Threshold Details

At the 0.85 threshold, which supplements the confidence–coverage curve in the main manuscript, the two-stage model achieved 95.34% selective accuracy at 58.98% coverage. At 0.95, the direct model achieved 97.09% selective accuracy on 1100 images, and the two-stage model 97.28% on 1102 images; review counts were 1847 and 1845, respectively. The difference of approximately 0.19 percentage points was treated descriptively; no paired confidence interval was calculated at this threshold.

The same confidence threshold retains different numbers of decisions across models. An additional comparison based on equal numbers of retained decisions and reviews was therefore calculated from stored outer-test probabilities (S6.2). Confidence describes the classification response. Endpoints were review-candidate counts and model errors relative to source labels; the success of expert correction is a separate target for prospective evaluation.

S2.4. Sperm-Identity Threshold Sweep

The sperm-identity threshold was varied using stored outer-test probabilities without retraining. The decision rule assigned an image to sperm when $p(S) \geq t$ and to non-sperm when $p(S) < t$. In the two-stage model, $p(S)$ was the calibrated first-stage sperm identity probability; in the direct model, it was the sum of normal and abnormal probabilities. The threshold was varied from 0.00 to 1.00 in increments of 0.01, and bidirectional error counts were calculated at 101 points on the same 2947 images. This binary identity decision differs from the main experiment's three-class rule, which selects the class with the highest final probability. At the 0.50 identity threshold, erroneous acceptance and exclusion counts were 83 and 123 for the two-stage model and 106 and 69 for the direct model. Lowering the two-stage threshold to 0.40 changed these counts to 96 and 93, respectively. In the main experiment's three-class decisions, corresponding counts were 77 and 141 for the two-stage model and 94 and 82 for the direct model. Identity ROC-AUC values were 0.978 and 0.979, respectively. Sweep points maximizing Youden's J statistic, defined as sensitivity minus false-positive rate, were 0.48 and 0.60 for the two-stage and direct models; points where the two class-specific error rates were closest were 0.58 and 0.71. These were reported as descriptive summaries of the threshold–error relationship in stored test predictions; the main experiment's decision rule was retained and results were evaluated separately. Counts and rates at selected thresholds are presented in Table S4, and a graphical summary of the full range is provided in Figure 8 of the main manuscript. Detailed results for all 101 threshold points can be generated using the threshold_sweep.py script in the reproducibility package [49].

Table S4. Bidirectional error counts and class-specific rates at selected sperm-identity thresholds. Type 1 denotes erroneous acceptance of a non-sperm image as sperm; Type 2 denotes exclusion of a reference sperm image as non-sperm. The respective denominators are 973 non-sperm and 1974 sperm images. These results are based on a binary identity threshold; error counts for the main experiment's final three-class decisions are reported in Section 3.4 of the main manuscript.

Model	Threshold (t)	Type 1 count	Type 2 count	Type 1 rate (%)	Type 2 rate (%)
Two-stage	0.30	116	76	11.92	3.85
Two-stage	0.40	96	93	9.87	4.71
Two-stage	0.48	83	115	8.53	5.83

Two-stage	0.50	83	123	8.53	6.23
Two-stage	0.58	73	149	7.50	7.55
Two-stage	0.60	70	155	7.19	7.85
Two-stage	0.70	55	200	5.65	10.13
Direct	0.30	160	36	16.44	1.82
Direct	0.40	137	48	14.08	2.43
Direct	0.48	114	61	11.72	3.09
Direct	0.50	106	69	10.89	3.50
Direct	0.58	84	88	8.63	4.46
Direct	0.60	80	96	8.22	4.86
Direct	0.70	69	131	7.09	6.64

S3. Discrimination Curves

Final-class ROC and PR curves were calculated on 2947 images using a one-versus-rest approach. Positive counts were 1002 for normal, 972 for abnormal, and 973 for non-sperm. In Stage 1, 1974 of 2947 images constituted the sperm positive class. In Stage 2, the denominator was restricted to 1974 reference sperm images, and the positive class comprised 972 abnormal images.

S3.1. ROC Results

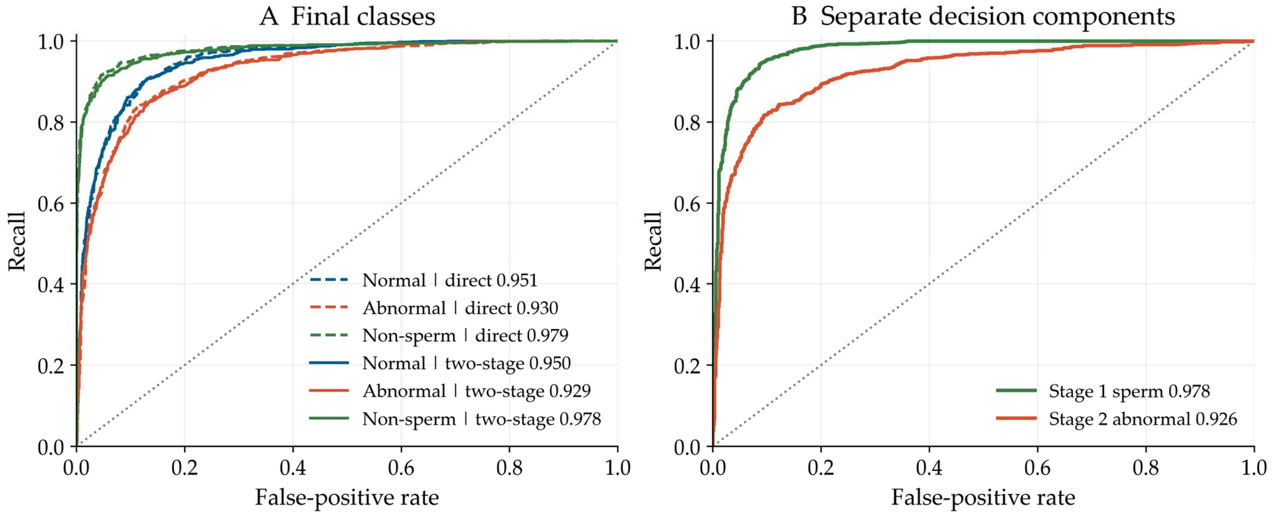

Figure S1. ROC curves from the main experiment. A, one-versus-rest discrimination for final classes; dashed lines indicate the direct model and solid lines the two-stage model. B, discrimination of sperm identity and abnormal morphology within the reference sperm subset. Legend values are ROC-AUC. The diagonal indicates random ranking. ROC, receiver operating characteristic; AUC, area under the curve.

ROC-AUC values for the two-stage and direct models were 0.950 and 0.951 for normal, 0.929 and 0.930 for abnormal, and 0.978 and 0.979 for non-sperm, respectively. Ranking performance was similar between decision structures. AUC was 0.978 for the sperm-identity component and 0.926 for the morphology component within reference sperm images. Component AUC values summarize within-task discrimination for sperm identity and morphology in the reference sperm subset.

S3.2. Precision–Recall Results

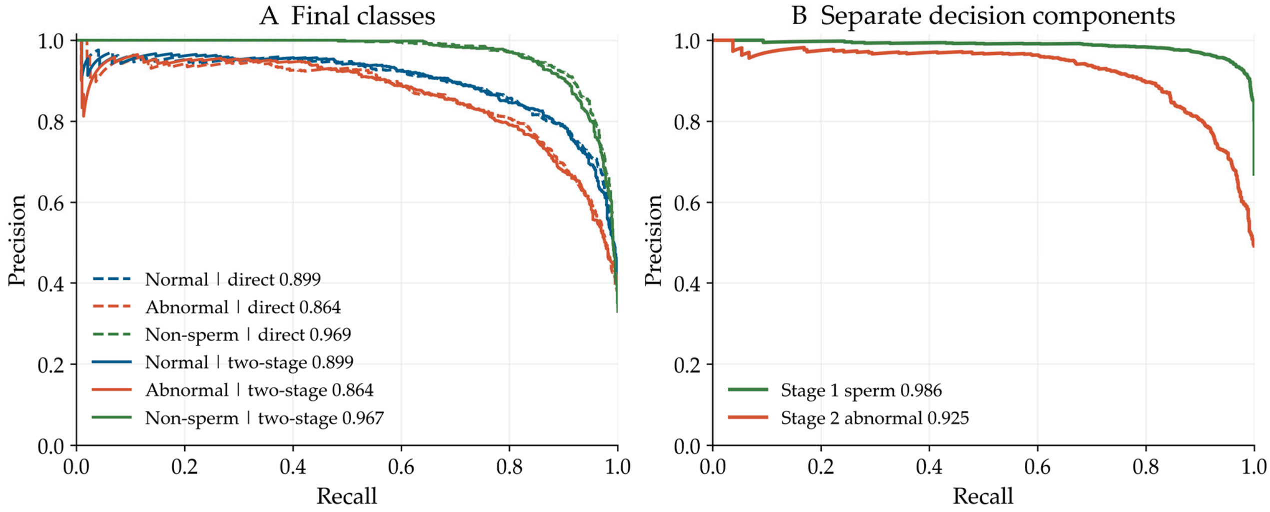

Figure S2. PR curves from the main experiment. A, one-versus-rest evaluation of the three final classes. B, results with sperm as the positive class in Stage 1 and abnormal as the positive class in Stage 2. Legend values are AP. AP summarizes the precision–recall relationship across thresholds. PR, precision–recall; AP, average precision; ROC-AUC, area under the receiver operating characteristic curve.

In Figure S2A, AP values for the two-stage model were 0.899 for normal, 0.864 for abnormal, and 0.967 for non-sperm; corresponding values for the direct model were 0.899, 0.864, and 0.969. Lower AP for the abnormal class indicates that detection of more abnormal images should be considered alongside the proportion of abnormal predictions agreeing with the source label. Correct detection of abnormal images and erroneous abnormal decisions for normal or non-sperm images should be evaluated together.

In Figure S2B, AP was 0.986 for Stage 1 and 0.925 for Stage 2. Positive-class proportions were 66.98% and 49.24%, respectively, whereas the final abnormal class comprised 32.98% of the entire archive. Because PR metrics depend on class prevalence, AP values were interpreted alongside the positive-class proportion for each task. Morphologically, errors in normal–abnormal discrimination among reference sperm images warrant separate examination. Archive class proportions do not represent the cell distribution within an ejaculate.

S4. Probability Calibration

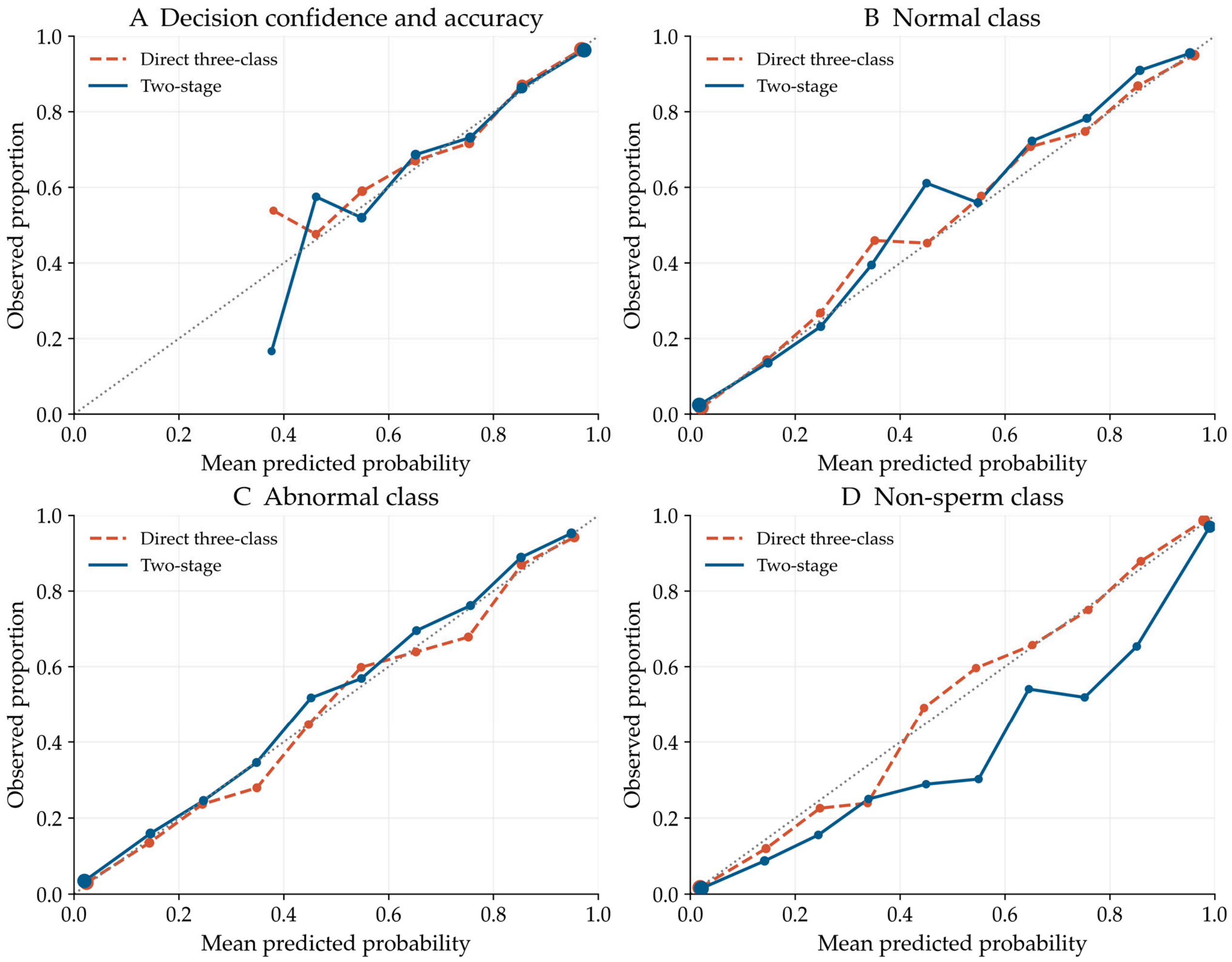

Figure S3. Calibration plots from the main experiment. A, final decision confidence versus observed accuracy. B–D, class probability versus observed class proportion. Each point summarizes a probability bin of width 0.10; point size increases with sample count. Predicted and observed proportions are equal on the diagonal. No pre- versus post-calibration comparison was performed.

In Figure S3A, the two-stage model's 1455 images in the 0.90–1.00 confidence interval had a mean confidence of 97.28% and observed accuracy of 96.29%. For the 519 images in the 0.80–0.90 interval, these values were 85.42% and 86.32%. Only 12 images fell in the 0.30–0.40 interval, so the observed proportion at that point was based on a small sample. Overall expected calibration error (ECE) was 0.019; bin-level and class-specific plots show local probability agreement that complements this aggregate value.

Among 75 images with a non-sperm probability of 0.80–0.90, the mean probability was 85.12%, and the observed non-sperm proportion was 65.33%; 26 images were labeled as sperm in the source (Figure S3D). Images in this interval may warrant separate examination during further validation for erroneous exclusion of true sperm. Cell integrity, tail visibility, and image quality can be considered together in expert assessment; results in this interval were presented to describe observed errors.

S5. Conformal Evaluation

S5.1. Development, Calibration, and Test Partitions

As shown in Table S5, 471 similarity groups comprising 471–473 images were reserved for conformal calibration in each fold. Model development used 1885–1887 images, including 1259–1266 reference sperm images for Stage 2. Test sizes remained the same as in the main experiment. Within each outer fold, no similarity group occurred in more than one of the development, calibration, and test partitions. The prediction-set threshold was therefore determined using data separate from model selection and temperature scaling.

Table S5. Separate data partitions in the supplementary conformal experiment.

Outer fold	Development images	Development sperm images	Calibration images	Calibration groups	Test images
1	1885	1263	472	471	590
2	1885	1259	472	471	590
3	1885	1266	473	471	589
4	1887	1266	471	471	589
5	1885	1265	473	471	589

In the supplementary arm retrained with a separate calibration partition, point-prediction accuracy was 84.22% for the direct model and 83.68% for the two-stage model. These results are reported separately from the main experiment; conformal prediction sets indicate possible class labels that cannot be excluded for each image.

S5.2. Coverage and Review Candidates

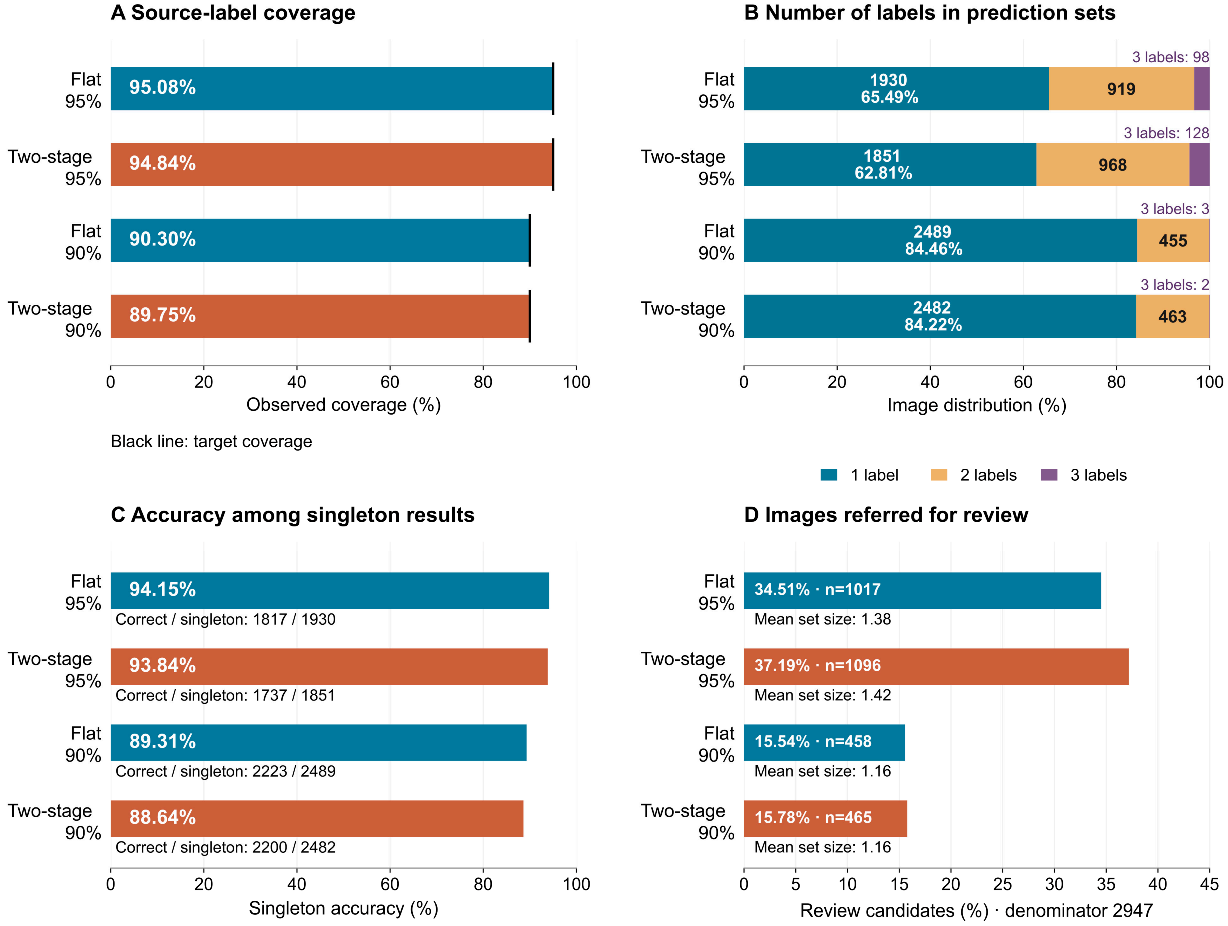

Figure S4. Conformal experiment retrained with separate calibration. A, proportion of sets containing the source label; black lines indicate target coverage. B, distribution by number of labels; singleton bars show counts and proportions, and other bars show counts. C, singleton accuracy with correct/total counts. D, review-candidate proportion and count, and mean number of labels across all images. Results with two or three labels are review candidates; no empty sets occurred. 'Direct' denotes the three-class comparator, and percentages in group labels indicate target coverage. Each group contains 2947 images. The 'Flat' label in the figure denotes the direct three-class model.

In Figure S4, at the 95% target, two-stage prediction sets contained the source label for 2795 of 2947 images (94.84%). Of 1851 singleton results, 1737 were correct and 114 incorrect, yielding 93.84% accuracy in this subset. At the same target, the direct model achieved 95.08% coverage and 94.15% singleton accuracy. Group-level coverage was 94.87% for the two-stage model and 95.11% for the direct model. Target coverage, observed coverage, and singleton accuracy are distinct metrics.

Increasing the two-stage target from 90% to 95% raised the review-candidate count from 465 to 1096. An additional 631 images were referred, while singleton accuracy increased from 88.64% to 93.84%. At the same targets, direct model review counts were 458 and 1017. These findings indicate that increasing target coverage increases both singleton accuracy and review volume.

At the 95% target, mean set size was 1.42 for the two-stage model and 1.38 for the direct model; at the 90% target, both decreased to 1.16. Singleton rates at the 95% target were 62.81% and 65.49%, respectively. Prediction-set size indicates classes that cannot be excluded under the calibration threshold and therefore model class uncertainty; it does not indicate the number of histological diagnoses.

S5.3. Class-Specific Coverage

Table S6. Class-specific coverage and review candidates at the 95% target in the conformal experiment.

Reference class	n	Direct coverage (%)	Two-stage coverage (%)	Two-stage singleton n	Two-stage review n (%)
Normal	1002	96.11	95.01	537	465 (46.41)
Abnormal	972	93.62	92.70	471	501 (51.54)
Non-sperm	973	95.48	96.81	843	130 (13.36)

Coverage of the abnormal class was lower than that of the normal and non-sperm classes in both models. Two-stage referral rates were 51.54% for abnormal, 46.41% for normal, and 13.36% for non-sperm images (Table S6). This distribution showed that review workload was unequal across cell groups. These counts indicate how many images in each source class were candidates for expert review.

Sets containing both normal and abnormal labels identify candidates for reassessment of morphological class; sets containing non-sperm and a sperm label identify candidates for reassessment of cell identity. Three-label sets indicate broader class uncertainty. These labels do not assign multiple histological diagnoses to the same image. They indicate whether an expert might reassess cell identity or morphological class.

S6. Additional Comparisons and Reproducibility

S6.1. Same-Task Comparisons of Component Probabilities

Sperm-identity and conditional-morphology probabilities derived from the direct model were compared with separately learned components on the same tasks and denominators (Table S7). Sperm-identity accuracy was 94.06% for derived probabilities and 93.01% for the separate component; conditional-morphology accuracy was 85.21% and 85.46%, respectively. This comparison distinguishes expressing probabilities at two assessment levels from learning those levels separately. The numerical difference of 0.25 percentage points in conditional morphology was considered alongside overall classification and review outcomes in the same experiment.

Table S7. Component comparisons on the same tasks and denominators. Accuracy is expressed as a percentage. ROC-AUC measures discrimination, and the Brier score measures probability error.

Probability source	Task	n	Accuracy	ROC-AUC	Brier
Derived from direct model	Sperm	2947	94.06	0.979	0.045
Derived from direct model	Conditional abnormal	1974	85.21	0.924	0.107
Separately learned	Sperm	2947	93.01	0.978	0.052
Separately learned	Conditional abnormal	1974	85.46	0.926	0.105

S6.2. Comparison at Matched Review Volume

At each review volume, both models retained decisions for the same number of images; identities of the retained images could differ between models (Table S8; Figure S5). When each model referred 973 images, 109 incorrect decisions for the direct model and 125 for the two-stage model remained outside review. Referred incorrect decisions numbered 339 and 347, respectively, from initial error totals of 448 and 472. The lower number of remaining errors for the direct model at matched volume indicates that review strategies should be compared in terms of remaining

errors as well as referred errors. Results quantify model errors referred for review and remaining outside review relative to source labels.

Table S8. Accuracy and error counts among retained decisions at matched review counts. Models are listed consecutively for each coverage level.

Model	Review n	Retained n	Accuracy %	Remaining errors	Referred errors
Direct	1473	1474	96.68	49	399
Two-stage	1473	1474	96.20	56	416
Direct	1179	1768	95.42	81	367
Two-stage	1179	1768	94.97	89	383
Direct	973	1974	94.48	109	339
Two-stage	973	1974	93.67	125	347
Direct	925	2022	94.11	119	329
Two-stage	925	2022	93.42	133	339
Direct	884	2063	93.70	130	318
Two-stage	884	2063	93.21	140	332
Direct	589	2358	90.88	215	233
Two-stage	589	2358	90.46	225	247
Direct	295	2652	88.16	314	134
Two-stage	295	2652	87.48	332	140

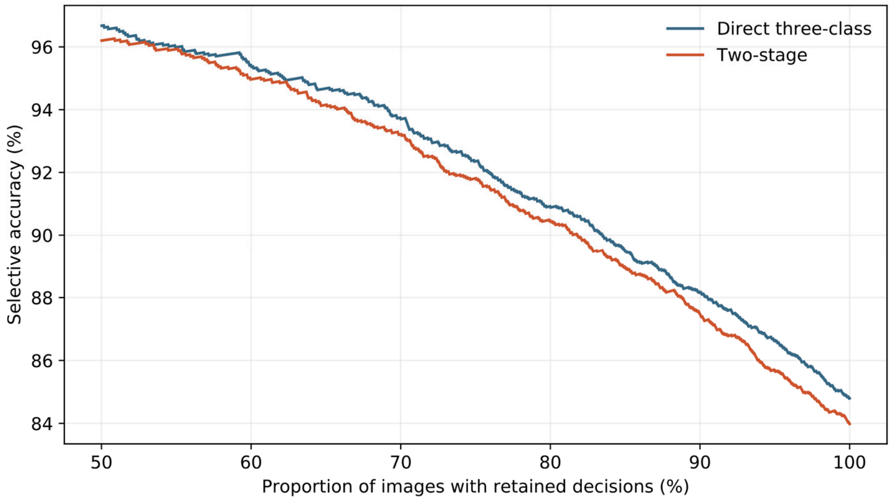

Figure S5. Selective accuracy at matched coverage. At each point, the same number of highest-confidence decisions is retained; coverage is shown over the 50–100% range. Curves were generated from stored outer-test predictions in the main experiment. No new threshold was selected from this exploratory plot.

S6.3. Class-Weight Sensitivity Analysis

The balanced weighting rule is n/(K × n_k), based on class counts in each training partition. The three-class and conditional-morphology tasks are approximately balanced, whereas sperm identity has an approximately 2:1 distribution. Unweighted training, with features, outer folds, inner model selection, and temperature calibration unchanged, tested the effect of this choice. Unweighted accuracy was 84.87% for the direct model and 84.39% for the two-stage model. The two-stage model produced 85 non-sperm-to-sperm errors and 118 sperm-to-non-sperm errors (Table S9). Together with 77 and 141 errors under balanced weighting, these findings indicate that class weights affect the magnitude of the trade-off. Overall accuracy was higher for the direct model under both weighting conditions. Primary balanced and supplementary unweighted results are reported separately. The maximum absolute probability difference between balanced retraining and the original records was below 6×10^{-14}.

Table S9. Effect of class weights within the same outer-test partitions. D, non-sperm; S, normal or abnormal sperm.

Weighting	Model	Accuracy %	Macro-F1	D → S errors	S → D errors
Balanced	Direct	84.80	0.848	94	82
Balanced	Two-stage	83.98	0.839	77	141
Unweighted	Direct	84.87	0.849	94	81
Unweighted	Two-stage	84.39	0.843	85	118

S6.4. Comparison with Published Methods

Table S10 summarizes dataset targets, methods, and reported results of relevant studies. Previous publications focus on image representations, feature processing, and anatomical class design. Here, shared representations and identical test records were used to examine how separate conditional learning changed bidirectional identity errors and decisions at matched review volume. Performance metrics in the table are presented alongside each publication's data and validation conditions. Paired comparisons in this study used the same audited dataset and the same outer-test folds. Results from AlSaad et al. [23] are reported as macro-F1. The source-balanced mean was 0.91, and the SMIDS subset value was 0.89. Their task was binary normal–abnormal classification of sperm images from three sources.

Table S10. Methodological context of relevant studies. Accuracy and macro-F1 are reported alongside each publication's task and validation design. CNN, convolutional neural network; CBAM, convolutional block attention module; SVM, support vector machine.

Study	Data and task	Method	Reported result	Evaluation context
Ilhan et al. [20]	SMIDS; 3 classes	MobileNet; detection and classification	87%	Source study; different image-processing pipeline
Aktas et al. [21]	SMIDS and HuSHeM	BEiT-Base; CNN and vision transformer comparison	SMIDS 92.5%	Focus on architecture and augmentation; study-specific validation
Kılıç [22]	SMIDS; 3 classes	ResNet50 + CBAM; feature selection and SVM	96.08%	Five-fold evaluation reported; different preprocessing and feature selection
Turkoglu et al. [24]	18 morphology classes; 3 stains	Two-stage ensemble based on anatomical groups	69.43% / 71.34% / 68.41%	Classification of anatomical morphology subclasses

AlSaad et al. [23]	SMIDS, HuSHeM, Hi-LabSpermMorpho; binary normal–abnormal task	Fully fine-tuned SigLIP2; source-specific evaluation	Macro-F1, source-balanced mean 0.91; SMIDS 0.89	Binary task excluding non-sperm; study-specific test partitions
Yang et al. [33]	Live sperm; head, midpiece, and principal-piece morphology	EfficientNet-B0 integrated with tracking and segmentation	Morphological accuracy 90.82%	Full-system result; scope differs from the static three-class SMIDS task
This study	SMIDS; 2947 images after audit	Frozen EfficientNet-B0; direct and conditional logistic regression	84.80% / 83.98%	Same 5 outer/3 inner group folds; error direction and matched review volume

S6.5. Records and Reproducibility Package

The reproducibility package has been uploaded to Zenodo (DOI 10.5281/zenodo.23211652) [49]. In the package, recorded_outputs/data_manifest.csv links record identifiers, source paths, original classes, labels, SHA-256 image hashes, and similarity groups; recorded_outputs/outer_fold_predictions.csv links each record to its outer-test fold and original outer-test probabilities. Additional component comparisons, matched-review-volume results, class-weight sensitivity tables, and feature-extractor comparisons are generated by scripts in the training/revision folder. The package includes the code and SHA-256 checksums of its files (SHA256SUMS.json; verify.py); the code is released under the MIT License, SMIDS images are distributed under CC BY 4.0 by their original repository [25], and EfficientNet weights follow their providers' terms. The training/single_cell_gradcam.py script generates numerical Grad-CAM maps and records SHA-256 hashes of source images for the Grad-CAM example.

S6.6. Sensitivity to Feature-Extractor Choice

B0 was retained as the main experiment. In the supplementary evaluation, B1 and B2 were assessed as frozen feature extractors using ImageNet IMAGENET1K_V1 weights. The same 2947 images, 2942 similarity groups, five outer and three inner folds, 224 × 224 preprocessing, balanced logistic regression, and training-data-only temperature calibration were used. B0 and B1 produced 1280 features, and B2 produced 1408. Direct and two-stage models were evaluated for each extractor. Retraining reproduced the main B0 predictions with an absolute probability difference below 10^{-8}. Sensitivity to feature-extractor choice was evaluated with network weights and the 224 × 224 input configuration held fixed. This design allowed comparison of the two learning approaches under identical preprocessing and test conditions.

Table S11. Overall accuracy, identity errors, and matched-review-volume results by feature extractor. Each model referred 973 images for review, leaving 1974 images for evaluation. Error counts refer to final three-class decisions.

Model	Learning	Overall accuracy (%)	Non-sperm → sperm (n)	Sperm → non-sperm (n)	Retained accuracy (%)	Remaining errors (n)
B0	Direct	84.80	94	82	94.48	109
B0	Two-stage	83.98	77	141	93.67	125
B1	Direct	85.21	84	66	94.17	115
B1	Two-stage	84.83	56	117	94.02	118
B2	Direct	83.68	92	88	93.97	119
B2	Two-stage	83.37	61	158	93.62	126

On the 1974 reference sperm images, normal–abnormal accuracy of the second component was 85.46%, 84.95%, and 84.35% for B0, B1, and B2, respectively. Corresponding values for conditional-morphology probabilities derived from the direct model were 85.21%, 84.50%, and 83.43%. B1 had the numerically highest overall accuracy, whereas B0 had the highest conditional-morphology accuracy. Across all three feature extractors, the two-stage model reduced non-sperm-to-sperm errors and increased errors in the reverse direction. Overall accuracy and selective accuracy when 973 images were referred were higher for the direct model in all three cases.

Statistical comparisons between feature extractors were exploratory analyses planned after the results were observed. B0–B1, B0–B2, and B1–B2 were compared for direct and two-stage three-class decisions and for conditional-morphology decisions under both learning structures. Morphology was evaluated only on reference sperm images at a decision threshold of 0.5. The primary comparison used a two-sided group randomization test that jointly flipped the signs of paired accuracy differences within each similarity group (100,000 iterations). An image-level two-sided exact McNemar test was calculated as a sensitivity analysis. Holm correction was applied across all 12 comparisons within each test family. The 95% confidence intervals were pointwise percentile intervals obtained by sampling similarity groups with replacement 10,000 times; they were not adjusted for multiple comparisons. Models were not retrained during resampling.

Table S12. Paired accuracy differences between feature extractors. Differences are the first model minus the second, expressed in percentage points. Confidence intervals are pointwise; Holm correction across 12 comparisons was applied separately to each p-value column.

Task	Pair	Difference	95% confidence interval	Group test p	McNemar p
Direct three-class	B0-B1	−0.41	−1.73 to 0.95	1.000	1.000
Direct three-class	B0-B2	1.12	−0.20 to 2.44	0.987	0.957
Direct three-class	B1-B2	1.53	0.14 to 2.88	0.395	0.392
Two-stage three-class	B0-B1	−0.85	−2.17 to 0.54	1.000	1.000
Two-stage three-class	B0-B2	0.61	−0.75 to 2.00	1.000	1.000
Two-stage three-class	B1-B2	1.46	0.07 to 2.82	0.427	0.426
Direct conditional morphology	B0-B1	0.71	−0.96 to 2.38	1.000	1.000
Direct conditional morphology	B0-B2	1.77	0.10 to 3.40	0.427	0.426
Direct conditional morphology	B1-B2	1.06	−0.66 to 2.84	1.000	1.000
Second-component morphology	B0-B1	0.51	−1.06 to 2.08	1.000	1.000
Second-component morphology	B0-B2	1.11	−0.46 to 2.74	1.000	1.000
Second-component morphology	B1-B2	0.61	−1.06 to 2.33	1.000	1.000

All Holm-adjusted p-values in the paired feature-extractor comparisons exceeded 0.05. For the second component, the B0–B1 difference was 0.51 percentage points (95% confidence interval, −1.06 to 2.08), and the B0–B2 difference was 1.11 percentage points (−0.46 to 2.74). This analysis evaluated sensitivity to feature-extractor choice within the same image-similarity groups. Absence of a significant difference was not interpreted as equivalence. Grad-CAM panels came from the B0-based main experiment, whereas conformal results came from the B0-based supplementary experiment retrained with separate calibration. The B1–B2 comparison covers overall classification, component accuracy, identity errors, and matched-review-volume results.

References

Reference numbers correspond to those in the main manuscript.

5. World Health Organization (WHO). WHO Laboratory Manual for the Examination and Processing of Human Semen, 6th ed.; World Health Organization: Geneva, Switzerland, 2021. Available online: https://www.who.int/publications/i/item/9789240030787 (accessed on 17 September 2026).

20. Ilhan, H.O.; Sigirci, I.O.; Serbes, G.; Aydin, N. A fully automated hybrid human sperm detection and classification system based on mobile-net and the performance comparison with conventional methods. Med. Biol. Eng. Comput. 2020, 58, 1047–1068. https://doi.org/10.1007/s11517-019-02101-y.

21. Aktas, A.; Serbes, G.; Ilhan, H.O. Unveiling the capabilities of vision transformers in sperm morphology analysis: a comparative evaluation. PeerJ Comput. Sci. 2025, 11, e3173. https://doi.org/10.7717/peerj-cs.3173.

22. Kılıç, Ş. Deep feature engineering for accurate sperm morphology classification using CBAM-enhanced ResNet50. PLoS ONE 2025, 20, e0330914. https://doi.org/10.1371/journal.pone.0330914.

23. AlSaad, R.; Albasha, S.; Burjaq, H.; AlBader, M.; Thomas, R. Evaluation of vision transformers and vision foundation models for sperm morphology analysis. Front. Reprod. Health 2026, 8, 1883326. https://doi.org/10.3389/frph.2026.1883326.

24. Turkoglu, A.K.; Serbes, G.; Uzun, H.; Aktas, A.; Yigit, M.H.; Ilhan, H.O. Category-Aware Two-Stage Divide-and-Ensemble Framework for Sperm Morphology Classification. Diagnostics 2025, 15, 2234. https://doi.org/10.3390/diagnostics15172234.

25. Ilhan, H. Sperm Morphology Image Data Set (SMIDS), Version 1; Mendeley Data, 2022. https://doi.org/10.17632/6xvdhc9fyb.1.

27. Guo, C.; Pleiss, G.; Sun, Y.; Weinberger, K.Q. On Calibration of Modern Neural Networks. In Proceedings of the 34th International Conference on Machine Learning; Proceedings of Machine Learning Research;, 2017; Volume 70, pp. 1321–1330. Available online: https://proceedings.mlr.press/v70/guo17a.html (accessed on 17 September 2026).

28. Selvaraju, R.R.; Cogswell, M.; Das, A.; Vedantam, R.; Parikh, D.; Batra, D. Grad-CAM: Visual Explanations from Deep Networks via Gradient-Based Localization. In Proceedings of the 2017 IEEE International Conference on Computer Vision (ICCV);, 2017; pp. 618–626. https://doi.org/10.1109/iccv.2017.74.

29. Angelopoulos, A.N.; Bates, S. Conformal prediction: A gentle introduction. Found. Trends Mach. Learn. 2023, 16, 494–591. https://doi.org/10.1561/2200000101.

30. Geifman, Y.; El-Yaniv, R. Selective Classification for Deep Neural Networks. Advances in Neural Information Processing Systems 2017, 30. https://proceedings.neurips.cc/paper/2017/hash/4a8423d5e91fda00bb7e46540e2b0cf1-Abstract.html

32. Raghu, M.; Zhang, C.; Kleinberg, J.; Bengio, S. Transfusion: Understanding Transfer Learning for Medical Imaging. Advances in Neural Information Processing Systems 2019, 32. https://proceedings.neurips.cc/paper/2019/hash/eb1e78328c46506b46a4ac4a1e378b91-Abstract.html

33. Yang, H.; Ma, M.; Chen, X.; Chen, G.; Shen, Y.; Zhao, L.; Wang, J.; Yan, F.; Huang, D.; Gao, H.; et al. Multidimensional morphological analysis of live sperm based on multiple-target tracking. Computational and Structural Biotechnology Journal 2024, 24, 176–184. https://doi.org/10.1016/j.csbj.2024.02.025.

49. Zayman, E.; İnceoğlu, F. Two Stage Probabilistic Classification of Sperm Identity and Morphology - Data Analysis, Version R2; Zenodo, 2026. https://doi.org/10.5281/zenodo.23211652.
